# Mite communities (Acari: Mesostigmata, Oribatida) in the red belt conk, *Fomitopsis pinicola* (Polyporales), in Polish forests

**DOI:** 10.1007/s10493-021-00635-1

**Published:** 2021-06-29

**Authors:** Anna K. Gdula, Piotr Skubała, Bogna Zawieja, Dariusz J. Gwiazdowicz

**Affiliations:** 1 Faculty of Forestry and Wood Technology, Poznan University of Life Sciences, Wojska Polskiego 71C, 60–625 Poznan, Poland; 2 Faculty of Natural Sciences, University of Silesia in Katowice, Bankowa 9, 40-007 Katowice, Poland; 3 Department of Mathematical and Statistical Methods, Poznan University of Life Sciences, Wojska Polskiego 28, 60–637 Poznan, Poland

**Keywords:** Microarthropods, Bracket fungi, Fruiting bodies, Natural forest, Anthropopressure

## Abstract

The fruiting bodies of bracket fungi are a specific microhabitat colonized by various invertebrates of which mites (Acari) are rarely studied, and if they are, the study is usually faunistic. The aim of the research was to determine whether the diversification of mite assemblages (Mesostigmata, Oribatida) inhabiting the fruiting bodies of *Fomitopsis pinicola* (Sw.) P. Karst. (Polyporales) are connected with the character of the forests and/or the degree of decay (DD) of the fruiting bodies. The research was conducted at Białowieża National Park (BNP), in forests close to natural ones and in Karkonosze National Park (KNP) which was affected by a large-scale forest dieback in the 1980s. Eighty fruiting bodies (40 at each study site) of *F. pinicola* belonging to four DD categories were collected. In total, 4,345 individuals of 120 mite species were recorded at BNP, and 13,912 individuals of 96 species were recorded at KNP. Analyses revealed that the sample dispersion at each study site was comparable, nevertheless the samples from each study site were clearly grouped into slightly overlapping sets which allow observation of the differences between them. In the less decayed fungi (DD 1 and 2) there were fewer mite species and individual mites than in the more decayed samples (DD 3 and 4). There were also significant differences between the fauna of the fungi in each particular DD: the fauna of DD 1 differed from all others, whereas the fauna of heavily decayed fungi (DD 3 and 4) was more comparable.

**Supplementary Information:**

The online version contains supplementary material available at 10.1007/s10493-021-00635-1.

## Introduction

Bracket fungi play a crucial role in the forest environment, they decompose wood and contribute to the effective processing of energy and matter flow in ecosystems (Niemelä 2013). Generally, the presence of these pathogens is undesirable in managed forests, as it generates significant economic losses, in the broader environmental perspective they contribute to a greater biological diversity (e.g., Lonsdale et al. 2008).

Bracket fungi create a specific microhabitat, i.e., wood decay, inhabited by numerous species representing many animal groups, starting with the invertebrates (Ehnström 2001; Evans et al. 2003; Kappes and Topp 2004), including mites (Skubała and Duras 2008; Gwiazdowicz et al. 2011; Huhta et al. 2012), then birds, e.g., woodpeckers (Conner et al. 1976; McClelland and McClelland 1999; Butler et al. 2004), and mammals (Suter and Schielly 1998; Bowman et al. 2000; Porter et al. 2005). Another, not so well studied, but highly unique microhabitat created by bracket fungi is a fruiting body, which has already been analysed for the presence of mites (Gwiazdowicz and Łakomy 2002; Maraun et al. 2014), insects (Jonsell and Nordlander 2004), spiders (Pielou and Pielou 1968), or arthropods in general (Pielou and Verma 1968; O'Connell and Bolger 1997a, b). This microhabitat is characterized by the presence of species which occur solely in the fruiting body of bracket fungi, such as mites from the genus *Hoploseius* (e.g., Walter 1998; Gwiazdowicz 2002; Mašán and Halliday 2016), e.g., *Hoploseius tenuis* (Lindquist 1965, 1995) with its elongated and narrow body. The research conducted so far (e.g., Walter 1998; Mašán and Halliday 2016) has revealed that the fruiting bodies of bracket fungi are inhabited by invertebrates that are characteristic for this microhabitat. Most previous research focused on the topic was faunistic in nature; hence, the factors that shape assemblage structure were not usually taken into consideration. However, Thunes and Willasen (1997) showed that the most significant variable for beetle communities was whether the inhabited fruiting body was dead or alive.

The red belt conk, *Fomitopsis pinicola* (Sw.) P. Karst. (Polyporales), is a widespread and a very common species of fungus which occurs worldwide in temperate and boreal forests in the Northern Hemisphere (Högberg et al. 1999; Shah et al. 2018). The fungus mainly attacks weaker and older trees in forests, parks and gardens, most often coniferous such as spruce (*Picea* spp.), pine (*Pinus* spp.) or fir (*Abies* spp.), and less frequently deciduous trees, e.g., birch (*Betula* spp.) and beech (*Fagus* spp.). It is known to cause a strong and fast-spreading brown rot which generates significant economic losses (Łakomy and Kwaśna 2008). Although *F. pinicola* is usually perceived in terms of losses in forestry due to wood depreciation caused by the fungus, it is also analysed from other viewpoints. Bače et al. (2012), for instance, analysed the species of fungus and its role in the seedling recruitment of logs in Central-European subalpine spruce forests, and also indicated that the presence of *F. pinicola* is negatively related to regeneration densities.

The Białowieża National Park (BNP), chosen for the study, is the largest area of natural forest in the North European Plain (Gutowski and Jaroszewicz 2004), and its pristine features have been well documented (Faliński 1986; Tomiałojć 1991; Jędrzejewska and Jędrzejewski 1998). As Białowieża Primeval Forest is located inside BNP, it is similar to natural forests, and has been a site for numerous acarological studies focusing on mite phoresy on beetles (Gwiazdowicz et al. 2013; Błoszyk et al. 2016), or parasitic Gamasides on mammals (Kozłowski and Żukowski 1958). In addition, there are many faunistic publications about the park (e.g., Rajski 1961; Olszanowski and Błoszyk 1998; Gwiazdowicz 2000). However, despite numerous acarological studies conducted at BNP, so far only one publication has been devoted to the mite presence in the fruiting bodies of bracket fungi in the national park (Gdula et al. 2021).

The second area that was analysed, Karkonosze National Park (KNP), constitutes an example of an area that has experienced a strong anthropogenic impact, particularly due to forest management, settlements, herding, and the exploitation of minerals (Danielewicz et al. 2002; Szymura et al. 2010). Among others, events which have had negative consequences for KNP are forest dieback caused by pollution and the accumulation of heavy metals in the soil and conifer needles (Sobik and Błaś 2008). The aftermaths of the disaster touched many species ranging from lichens, protozoa (Dąbrowska-Prot 1995), and avifauna (Jadczyk 2008). The acarofauna of KNP has been a subject of many studies (e.g., Gwiazdowicz and Biernacik 2000; Gwiazdowicz 2003); however, there are no studies dealing with mite assemblages inhabiting fruiting bodies of bracket fungi.

In this research focused on biodiversity in the two types of ecosystems, we tested the following hypotheses. (1) There are differences in density and structure of mite communities inhabiting the fruiting bodies of *F. pinicola* in the two diverse forest ecosystems: BNP, which is considered one of the largest natural forests in the North European Plain, and KNP, which is considered to be one of the most degraded forest complexes caused by an eco-disaster and acid rain. And (2) the structure of mite assemblages colonizing fruiting bodies of *F. pinicola* varied depending on their degree of decay (DD).

## Material and methods

### Study area

**Fig. 1:**
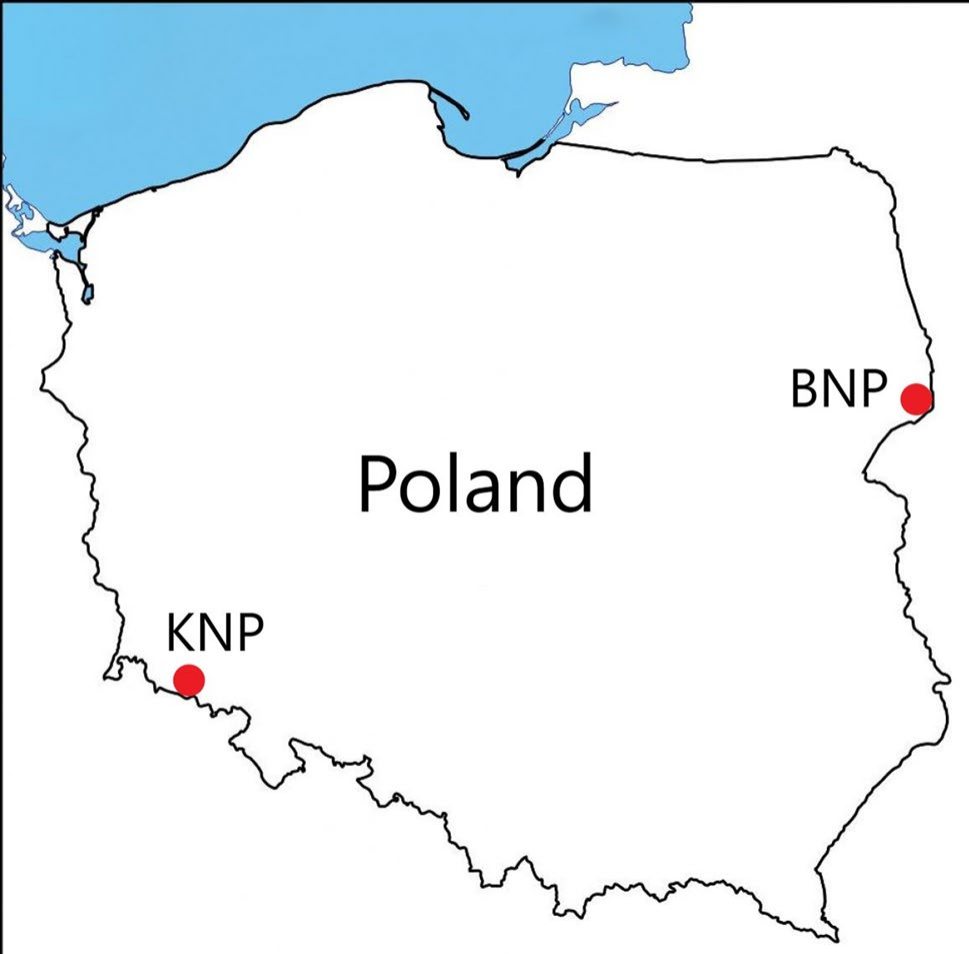
Location of the study areas (BNP – Białowieża National Park, KNP – Karkonosze National Park)

The Białowieża National Park (BNP) was established in 1947 which makes it the oldest Polish national park. BNP is located in the northeast of Poland (52°69′89′′ – 52°81′89 ′′ N, 23°71′76′′ – 23°93′95′′ E), near the village of Białowieża (Fig. 1). The main task of BNP is to protect one of the best-preserved primeval natural deciduous and mixed forests in Europe Lowland. In the Białowieża Primeval Forest, where the BNP is located, there are 27 forest communities with 24 tree species (Sokołowski 2004). The largest part of the BNP area (48.7%) is covered with the East-European oak-hornbeam forest *Tilio-Carpinetum* (*typicum*, *stachyetosum*, *caricetosum*, *circaeaetosum* and *calamagrostietosum*). In the park there are also marsh communities: valleys in a primeval forest, which are periodically flooded, covered with an ash-alder riparian forest *Circaeo-Alnetum* (8.4%), whereas peatland valleys and marshy interior basins are covered with alder swamp forests *Ribeso nigri-Alnetum* or *Sphagno squarrosi-Alnetum* (5.9%). Moreover, the boreal spruce forest on peatland *Sphagno girgensohnii-Piceetum* (1.6%) and the sub-boreal swampy birch forest *Thelypteridi-Betuletum pubescentis* (5.3%) are other communities that can be found there. The poorest habitats growing on sands are covered with pine forests: the subcontinental fresh coniferous forest *Vaccinio vitis-idaeae-Pinetum*, midland dry pine forests *Cladonio-Pinetum* (3.8%), swampy coniferous forest *Vaccinio uliginosi-Pinetum* and raised-bog community *Ledo-Sphagnetum magellanici* (1.7%) (Kwiatkowski et al. 2009; Jaroszewicz 2010). About 1500 species of Macromycetes fungi and about 240 species of lichens were found in BNP (Cieśliński 2009; Kujawa 2009). In 2008–2012, an inventory of polypore fungi was conducted in the BNP and 142 species were recorded (Niemelä 2013).

Karkonosze National Park (KNP) is a park in the Karkonosze Mountains in the Sudetes located in south-western Poland (50°73′59′′ – 50°84′10′′ N, 15°45′88′′ – 15°82′99′′ E), along the border with the Czech Republic in the highest part of the Sudetes (Fig. 1). In 1992, KNP, together with the neighboring Czech Krkonoše National Park, became a part of the Krkonoše/Karkonosze biosphere reserve under UNESCO’s Man and the Biosphere (MaB) programme. Amidst the plant communities occurring in KNP there are, among others: Central European oak-hornbeam forest *Galio sylvatici-Carpinetum betuli* and riverine alder forest *Alnetum incane* in foothill zone, acidophilic montane beech forest *Luzulo luzuloidis-fagetum* as well as lower mountain fir-spruce forest *Abieti-Piceetum* (*montanum*) in the lower mountain zone, and the upper mountain spruce forest (*Calamagrostio villosae-Piceetum*) in the upper mountain zone. In the subalpine zone there are Sudety dwarf-pine thickets *Pinetum mugo sudeticum* and downy willow shrub *Salicetum lapponum* (Danielewicz et al. 2002). The diversification of KNP fauna is connected with the occurrence of vegetation floors of different climates, a diverse mosaic of plant communities and the exploitation of natural resources in the past. The specification of KNP fauna consists of relatively numerous occurrences of alpine and boreal-mountain species, almost a total lack of thermophilic species and a significant specificity in comparison to other Polish mountains (Raj and Knapik 2014).

Due to the specifics of the research sites (forests in BNP and KNP are not replicated), the samples collected in each of the analyzed national parks may be considered as pseudoreplicates of one site.

### Material collection

The fruiting bodies of *F. pinicola* were collected on 18 and 19 July 2014 at the KNP and between 4 and 9 August 2014 at the BNP. In total, 80 fruiting bodies at different degrees of decay were collected on both locations (40 in BNP and 40 in KNP). An axe was used to remove them from tree trunks. The fruiting bodies were collected from the trees where *F. pinicola* occurred most often in the studied location, i.e., Norway spruce, *Picea abies*, which is a common woody species in both parks. In order to reflect the specificity of the studied locations, the samples were taken randomly according to the diameter at breast height and the age of the trees. The fruiting bodies were collected from tree trunks at the height between 0 cm (ground level) to 210 cm above ground level, taking into consideration their degree of decay (DD). A single fruiting body equals one sample.

The classification of the DDs of the fruiting bodies used in this study is borrowed from Gdula et al. (2021) and, like the wood decay scales (e.g., Maser et al. 1978), it is based on the differences in the occurrence of various features in the substrate with a different degree of decay: DD 1 – Fruiting body with fresh hymenophore, without visible signs of decay; DD 2 – Fruiting body with dry hymenophore, without visible signs of decay; DD 3 – Fruiting body with few traces of decomposition, e.g., single (up to 10) insect galleries; and DD 4 – Fruiting body with numerous traces of decay, such as insect galleries, easily crumbles.

### Laboratory procedures

In order to extract mesofauna from the bracket fungi they were placed for 72 h in Tullgren funnels. The collected mites were preserved in 70% ethanol, and divided into two taxonomic groups: mesostigmatid and oribatid mites. In order to identify the Mesostigmata, semi-permanent (using lactic acid) and permanent (using Hoyers medium) microslides were prepared. All adult and immature mesostigmatid mite individuals were examined using light microscope (Zeiss Axioskope 2) and identified using taxonomical literature (e.g., Karg 1993; Mašán 2001; Gwiazdowicz 2007, 2010).

The Oribatida (adults and immature individuals) were identified with a light microscope (Nikon Eclipse E600). Prior to the examination, the internal tissue had been removed using concentrated lactic acid, 60% lactic acid or lactophenol as a clearing agent, but with weakly sclerotized forms diluted lactic acid was also appropriate (Norton 1990). Oribatid mites were determined to the species level by following keys and original species descriptions (Olszanowski 1996; Weigmann 2006; Niedbała 2008). Classification was according to Weigmann (2006).

The mites were classified only to the higher taxonomic units, e.g., genus, but in statistical analyses they were treated as separate species. All material was deposited in the acarological collection at the Department of Forest Pathology, Poznań University of Life Sciences, Poland (Mesostigmata) and Institute of Biology, Biotechnology and Environmental Protection, University of Silesia, Poland (Oribatida).

### Statistical analysis

The assessment of statistical significance of the examined factors was based on: raw data collected in the box-plot graphs, the level of significance (p-value) obtained using the Mann–Whitney test (when comparing two species) and Kruskal–Wallis test (to compare DDs). A horseshoe effect has not been observed in the analysed experiment; hence, the samples collected at BNP and KNP together with the samples belonging to each particular DD from each study site were compared using principal coordinate analysis (PCoA). Bray–Curtis dissimilarity matrix was adopted as the distance matrix for PCoA. The weights that were used were a sum of observations for each particular site and divided by the sum of all observations (the data before the statistical analysis have been transformed by adopting Wisconsin Double transformation). To present the deployment of the DDs and parks on the graph, the sum of observations for each particular DD and park was used, and community data were analysed by two-way permutational ANOVA. Indicator species analysis was used to provide specific species for each park and DD. Cluster analysis following the Ward method for Bray–Curtis dissimilarity matrix was used for the parks and DDs. Next, the PCoA analysis was performed separately for Mesostigmata and Oribatida mites, which was followed by the permutation test of species selection and is most strongly correlated with PCoA1 and PCoA2 (multiple regression). This test pointed at the specific species for each given park. All testing was conducted at a significance level of 0.05. All analyses were conducted in the R environment using the procedures of the vegan package (Oksanen et al. 2019).

## Results

### Mite assemblages in the national parks

The total number of collected mite individuals in BNP was 4,345 (824 mesostigmatid and 3,521 oribatid mites), and the total number of species was 120 (37 mesostigmatid species and 83 oribatid species). The number of mite individuals per sample was between 1 and 622 (mean ± SE = 108.63 ± 22.63) – the number of Mesostigmata was between 0 and 101 (20.60 ± 3.61), whereas the number of Oribatida was between 0 and 580 (88.03 ± 21.06). The number of mite species per sample was between 1 and 38 (9.63 ± 1.26); the number of Mesostigmata species was between 0 and 10 (3.18 ± 0.38), and Oribatida species was between 0 and 28 (6.45 ± 1.00). The most numerous mite species in BNP were: *Carabodes femoralis* (2766 individuals), *Dendrolaelaps pini* (268) and *Carabodes subarcticus* (261). Among the 10 most numerous mite species in BNP, eight (including *D. pini*, *Hoploseius oblongus* and *Dinychus perforatus*) belonged to the Mesostigmata and two (*C. femoralis* and *C. subarcticus*) belonged to Oribatida.

**Fig. 2:**
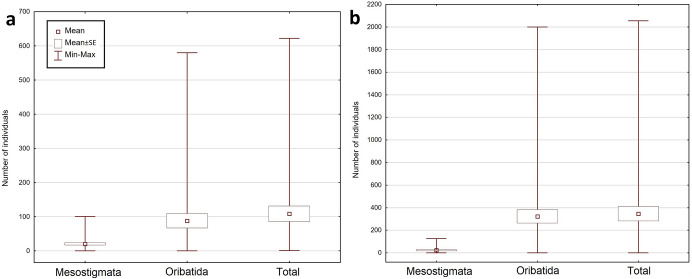
Minimum, maximum and mean (± SE) number of mite individuals (Mesostigmata, Oribatida, total) per sample, in **a** Białowieża National Park and **b** Karkonosze National Park

**Fig. 3:**
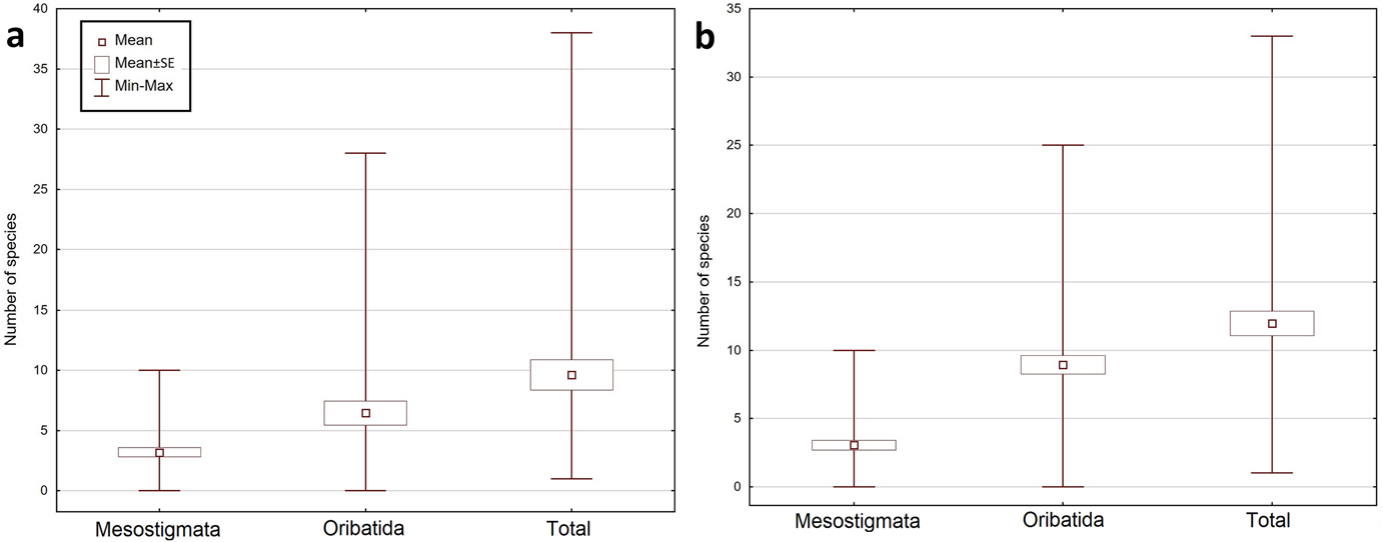
Minimum, maximum and mean (± SE) number of mite species (Mesostigmata, Oribatida, total) per sample, in **a** Białowieża National Park and **b** Karkonosze National Park

The total number of collected mite individuals in KNP was higher than in BNP – 13,912 (956 mesostigmatid and 12,956 oribatid mites), and the total number of mite species was lower: 96 (34 mesostigmatid and 62 oribatid species). The number of mite individuals per sample was between 1 and 2056 (mean ± SE = 347.80 ± 62.94), the number of Mesostigmata was between 0 and 126 (23.90 ± 4.96), whereas the number of Oribatida was between 0 and 2000 (323.90 ± 60.63). The number of mite species per sample was between 1 and 33 (11.98 ± 0.89); the number of Mesostigmata species was between 0 and 10 (3.05 ± 0.35), and the number of Oribatida was between 0 and 25 (8.93 ± 0.68). The most numerous mite species in KNP were: *Carabodes femoralis* (10,622 individuals), *Carabodes areolatus* (1030) and *Zerconopsis remiger* (256). The total share of Mesostigmata among the most numerous species in KNP was lower than in BNP: only four out of the 10 most numerous species (among others *Z. remiger*, *Hoploseius oblongus* and *Dendrolaelaps pini*) belonged to the Mesostigmata, and six (such as *C. femoralis*, *C. areolatus* and *Oribatella calcarata*) belonged to the Oribatida (Fig. 2, 3, Online Appendix).

The fungi collected at BNP contained considerably fewer mite individuals than at KNP. However, there was not only a greater number of species in the BNP sample, but also a different species composition. At both studied locations, the same species occurred in highest numbers (*Carabodes femoralis*); nevertheless, there were considerably fewer individuals (2766) in the samples from BNP than from KNP (10,622). Moreover, the composition of dominant species in each of the analysed mite group (Mesostigmata, Oribatida) in both national parks differed from one another. For instance, some of the most numerous Mesostigmata in BNP (like *Dendrolaelaps pini*) were significantly less numerous in KNP and outnumbered other species from their group (e.g., *Zerconopsis remiger*), whereas oribatid species such as the third most numerous species in BNP, *Carabodes subarcticus*, did not occur in KNP at all; *Carabodes areolatus*, the second most numerous species in KNP, occurred only rarely in the samples from BNP (Online Appendix).

**Fig. 4:**
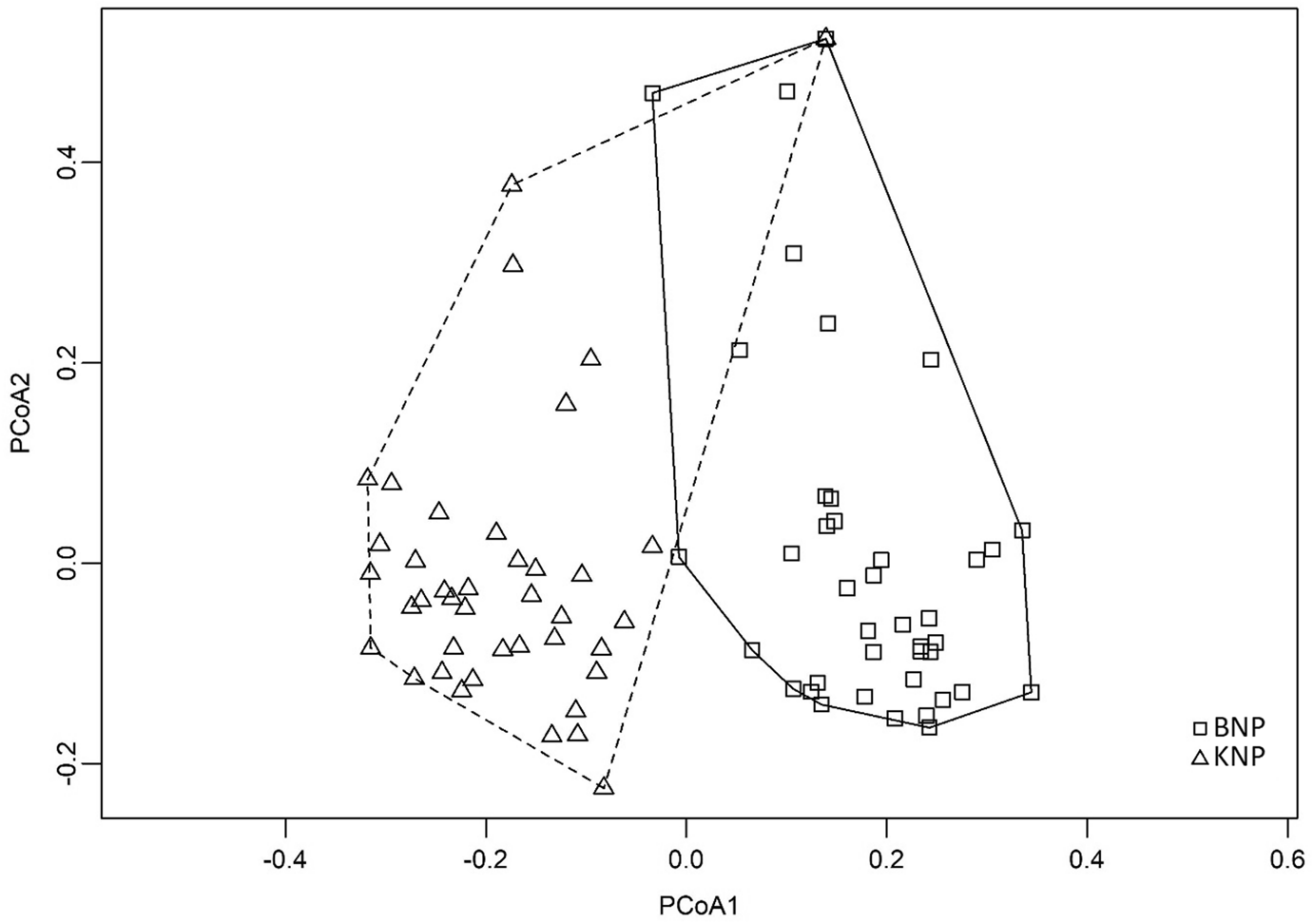
Principal coordinate analysis (PCoA) of the samples belonging to BNP (Białowieża National Park) and KNP (Karkonosze National Park)

The differences between the samples from the two national parks are emphasised by PCoA, for which the total Interia was 0.41. This indicated a clear grouping of the bracket fungi in each location into two corresponding sets, which only slightly overlap: the set of bracket fungi from KNP stretched along the lower values on the PCoA1 axis and the set of bracket fungi from BNP corresponded to higher values on PCoA1 axis (Fig. 4). Moreover, the permutation ANOVA indicated significant differences between the parks (p = 0.0001).

### Does the degree of decay of the bracket fungi determine the nature of mite assemblages?

**Fig. 5:**
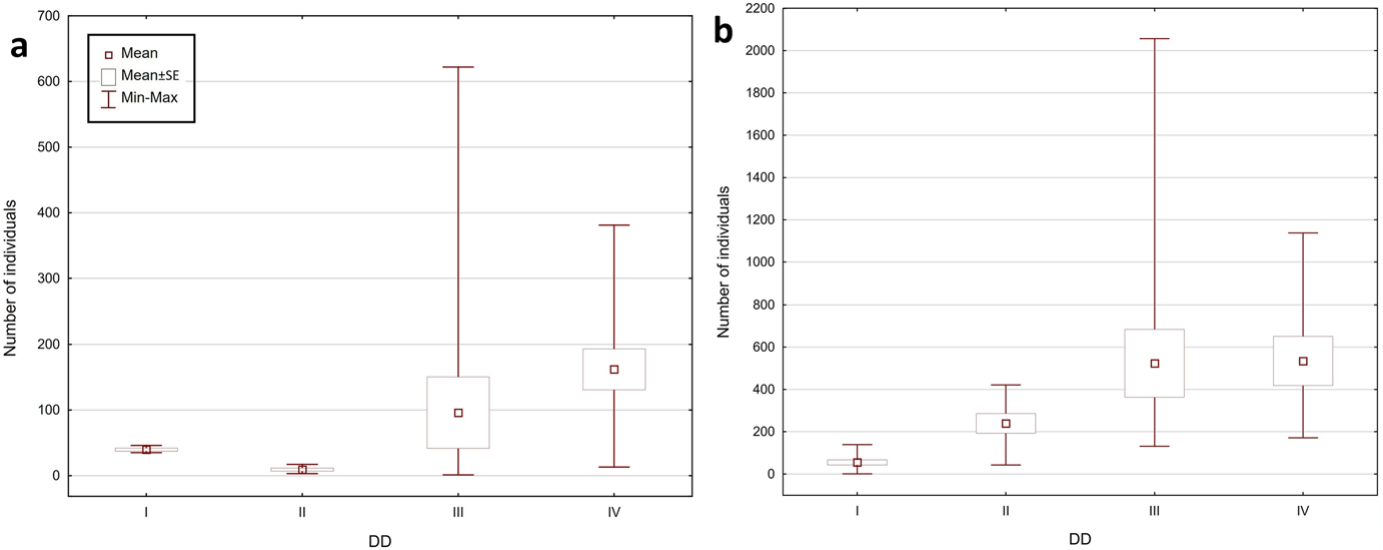
Minimum, maximum and mean (± SE) number of mite individuals per sample, depending on four degrees of decay (DD 1–4; see Table 1 for explanation) in **a** Białowieża National Park and **b** Karkonosze National Park

**Fig. 6:**
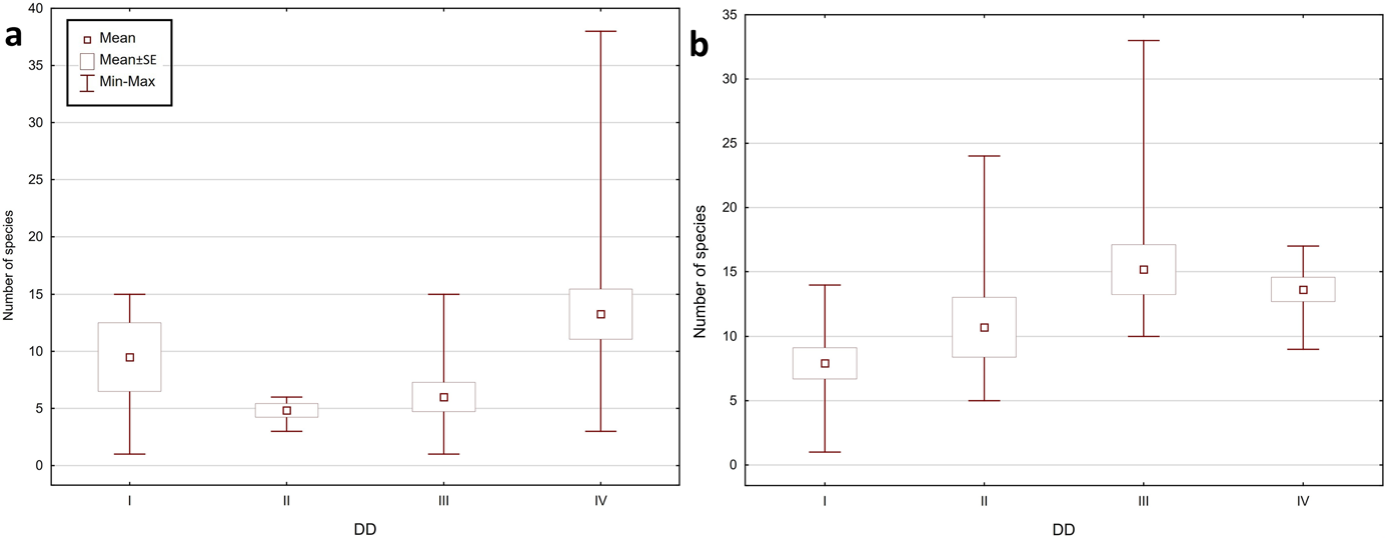
Minimum, maximum and mean (± SE) number of mite species per sample, depending on four degrees of decay (DD 1–4; see Table 1 for explanation) in **a** Białowieża National Park and **b** Karkonosze National Park

Significant differences were evident in BNP between the faunas inhabiting *F. pinicola* with different DD; however, there was no linear increase in the number of species and mite individuals as the decay of fruiting bodies increased. In the case of both the number of individuals and species, the lowest values were in the DD 2 samples, whereas the highest were in the DD 4 samples. In the least decayed fungi from KNP (DD 1), there were clearly fewer mite individuals, the numbers were slightly higher for the DD 2 samples, whereas the highest numbers of individuals were in the DD 3 and 4 samples. The lowest species richness in KNP was also noted in the least decayed bracket fungi (DD 1); however, the highest number of species occurred in DD 3 (Fig. 5, 6, Table 1, 2).

**Table 1:** Mean number (± SE; ranges in parentheses) of mite individuals per sample depending on the study site and four degrees of decay (DD)

		DD^a^			
		1	2	3	4
BNP	Mesostigmata	21.25 ± 5.33 (9–35)	2.33 ± 1.02 (0–7)	10.27 ± 3.71 (0–42)	32.21 ± 6.11 (1–101)
	Oribatida	18.25 ± 6.3 (0–28)	6.83 ± 2.18 (1–16)	85.73 ± 51.32 (0–580)	129.68 ± 30.08 (0–374)
	Total	39.5 ± 2.4 (35–46)	9.17 ± 2.43 (3–17)	96 ± 54.56 (1–622)	161.89 ± 31.4 (13–381)
KNP	Mesostigmata	12.82 ± 7.68 (0–88)	23.14 ± 9.44 (2–72)	24.73 ± 7.76 (0–85)	34.64 ± 13.1 (0–126)
	Oribatida	42 ± 9.25 (0–85)	215.71 ± 42.47 (28–357)	498.91 ± 157.21 (121–2000)	499.64 ± 108.66 (161–1012)
	Total	54.82 ± 12.46 (1–139)	238.86 ± 46.89 (42–421)	523.64 ± 160.44 (131–2056)	534.27 ± 116.33 (171–1138)

^a^DD 1 – fruiting body with fresh hymenophore, without visible signs of decay; DD 2 – fruiting body with dry hymenophore, without visible signs of decay; DD 3 – fruiting body with few traces of decomposition, e.g., single (up to 10) insect galleries; and DD 4 – fruiting body with numerous traces of decay, such as insect galleries, easily crumbles

*BNP*: Białowieża National Park, *KNP*: Karkonosze National Park

**Table 2:** Mean number (± SE; ranges in parentheses) of mite species per sample depending on the study site (BNP – Białowieża National Park, KNP – Karkonosze National Park) and four degrees of decay (DD)

		DD^a^			
		1	2	3	4
BNP	Mesostigmata	2 ± 0.41 (1–3)	1.5 ± 0.43 (0–3)	2.18 ± 0.42 (0–4)	4.53 ± 0.62 (1–10)
	Oribatida	7.5 ± 2.63 (0–12)	3.33 ± 0.71 (1–6)	3.82 ± 0.96 (0–11)	8.74 ± 1.8 (0–28)
	Total	9.5 ± 3.01 (1–15)	4.83 ± 0.6 (3–6)	6 ± 1.28 (1–15)	13.26 ± 2.2 (3–38)
KNP	Mesostigmata	1.45 ± 0.25 (0–3)	3.43 ± 1.19 (1–10)	3.91 ± 0.69 (0–8)	3.55 ± 0.56 (0–6)
	Oribatida	6.45 ± 1.13 (0–13)	7.29 ± 1.41 (3–14)	11.27 ± 1.61 (6–25)	10.09 ± 0.68 (7–13)
	Total	7.91 ± 1.22 (1–14)	10.71 ± 2.33 (5–24)	15.18 ± 1.92 (10–33)	13.64 ± 0.93 (9–17)

^a^See Table 1 for explanation

Differences were also evident in species composition of the mite assemblages at each of the study sites depending on the DD. The most numerous species of mesostigmatid mites in the bracket fungi of DD 1 (BNP) was *Hoploseius oblongus*, a species rare in the more decayed bracket fungi. In the fruiting bodies from DD 2, 3, and 4, the most numerous species was *Carabodes femoralis.* Differences were also evident among the most numerous species in each of DDs: in DD 1 bracket fungi only three out of 10 of the most recorded species belong to Mesostigmata, in DD 2 four species were mesostigmatid mites, whereas in DD 3 and 4 samples only six out of 10 of the most numerous species were Mesostigmata (Online Appendix).

The most numerous mite species in the samples of all DDs from KNP was *Carabodes femoralis*, but in the case of other species there were differences between the particular DDs. In the bracket fungi from DD 2, 3, and 4, the second most numerous species was *Carabodes areolatus*, which was considerably less frequent in the least decayed fungi. The second most numerous species in DD 1 was *Hoploseius oblongus*, which rarely occurred in the other DDs. In DD 1, nine out of 10 of the most numerous mite species was an Oribatida; among the 10 most numerous species in DD 2 bracket fungi, there were five species each of Mesostigmata and Oribatida. In DD 3 and 4, seven out of 10 most numerous mite species were Oribatida (Online Appendix).

**Fig. 7:**
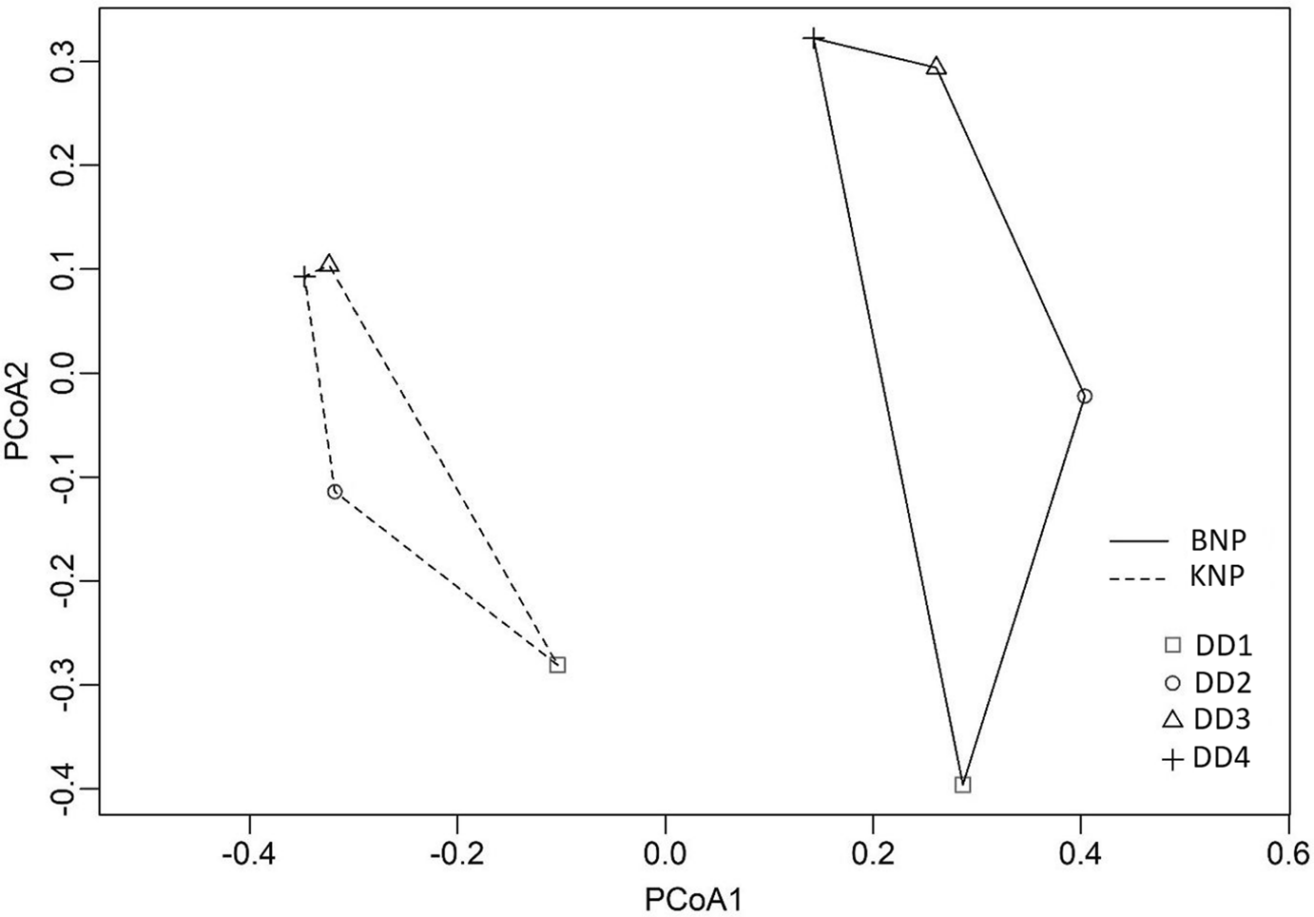
Principal coordinates analysis (PCoA) of the samples belonging to four degrees of decay (DD 1–4) in Białowieża National Park (BNP) and Karkonosze National Park (KNP)

The differences between the samples belonging to each particular DD group in the two parks were indicated by PCoA, whose total Inertia was 0.29. The analysis revealed that samples from particular study sites constituted concentrations which were dispersed to different degrees. In the case of bracket fungi from KNP, the set was less dispersed and matches the lower values on the PCoA1 axis, whereas the BNP set was more dispersed and shifted in the direction of higher values on the PCoA1 axis. The analysis also showed that the bracket fungi which were most similar were those from DD 3 and DD 4. Samples from DD 1 were further on the axis than others which signifies that they differ from the bracket fungi belonging to the higher DDs. PCoA also revealed some similarities in the manner of sample groupings in particular DDs in both parks; there was a trend in both KNP and BNP displaying that the least decayed bracket fungi, which differed from the samples of higher DDs, were located in the range of higher values on the PCoA2 axis. The bracket fungi from DD 3 and 4 in both study sites were similar to one another and group closely to the higher values on the axis (Fig. 7). The two-way permutational ANOVA (Table 3) of the community data (the assumption of homogeneity comparing groups was fulfilled; p = 0.050) also confirmed differentiation of the samples from BNP and KNP (p = 0.0001) and of the samples from various DDs (p = 0.0001). The interaction was significant as well (p = 0.017); therefore, the indicator species analysis was performed for parks and for DDs separately in each park. Twenty-nine selected species separated the two parks (p = 0.05). For KNP they were: *Carabodes areolatus*, *Zerconopsis remiger*, *Chamobates borealis*, *Cepheus dentatus*, *Oribatella calcarata*, *Scheloribates pallidulus*, *Phthiracarus longulus*, *Dendrolaelaps cornutus*, *Carabodes femoralis*, *Zercon storkani*, *Trachytes aegrota*, *Steganacarus* (*Atropacarus*) *striculus*, *Lasioseius zerconoides*, Parasitidae, *Oribatella quadricornuta*, *Carabodes reticulatus*, *Liebstadia pannonica*, *Carabodes tenuis*, *Pergamasus* sp.; for BNP they were: *Carabodes subarcticus*, *Zercon curiosus*, *Trichouropoda ovalis*, *Chamobates cuspidatus*, *Damaeus* sp., *Dinychus perforatus*, *Sejus togatus*, *Microgynium rectangulatum*, *Dendrolaelaps pini*, *Dinychus arcuatus*. Six species were selected in KNP. The DDs differed regarding five species: in DD 1, there were two indicator species: *Hoploseius oblongus*, and *Scheloribates pallidulus*; DD 2 also had two indicator species *Lasioseius ometes* and *Thenargamasus* sp.; in DD 4 one species was selected, namely Parasitidae. *Cepheus cepheiformis* was related to two groups: DD 3 and 4. The analysis conducted in BPN selected four indicator species, with *Parachipteria punctata* and *Lagenobates lagenulus* for DD 1, *Dinychus perforatus* for DD 4 and *Hoploseius oblongus* for DD 1 and 2.

**Table 3:** Permutational analysis of variance of the community data collected from two study sites (BNP – Białowieża National Park, KNP – Karkonosze National Park) and four degrees of decay (DD)

Factor	df	SS	MS	F	p
Study site (KNP, BNP)	1	3.3416	3.3416	10.8674	0.0010
DD	3	2.0303	0.6768	2.2009	0.0010
Study site * DD	3	1.2715	0.4238	0.04418	0.017
Residuals	72	22.1391	0.3075		

**Fig. 8:**
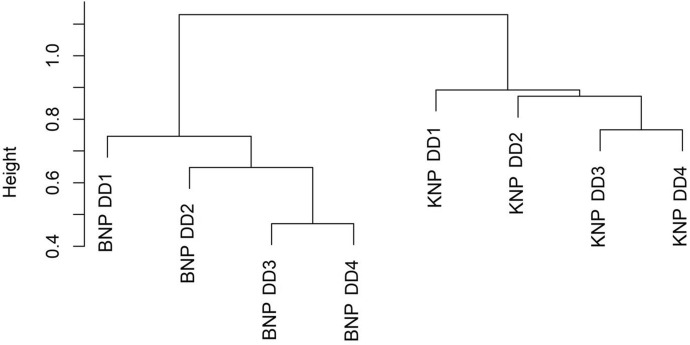
Cluster analysis (Ward method) of four degrees of decay (DD 1–4) in Białowieża National Park (BNP) and Karkonosze National Park (KNP)

Cluster analysis (Ward method) from Bray–Curtis dissimilarity matrix led to similar grouping of the bracket fungi as that based on PCoA. The most similar bracket fungi from BNP belonging to DD 3 and 4 constituted a cluster, to which the bracket fungi from DD 2 and 1 could be added provided they came from the same park. A separate cluster was created out of bracket fungi from KNP, among which the DD 3 and 4 bracket fungi were characterised by the greatest similarity, to which bracket fungi from DD 2 and fungi from DD 1 could be added (Fig. 8).

A separate analysis of the Mesostigmata and the Oribatida revealed similar trends, and confirmed the differences between the fruiting bodies belonging to various DDs in both locations. In the case of the mean number of individuals and the mean number of species for a single bracket fungus in BNP, in each DD oribatid mites outnumbered mesostigmatid mites. Only the samples for DD 1 were an exception as there were more Mesostigmata than Oribatida (mean ± SE = 21.25 ± 5.33 vs. 18.25 ± 6.3 per sample). However, there were more individuals and species of Oribatida in the samples from KNP in all DDs (Table 1, 2).

**Fig. 9:**
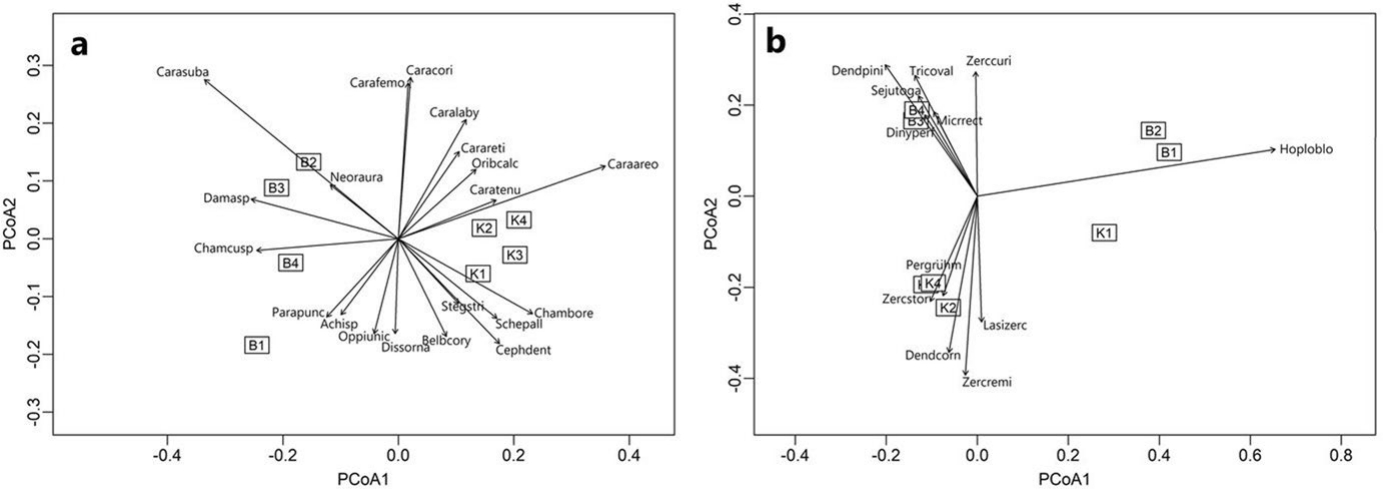
Principal coordinates analysis (PCoA) of the significant species and samples belonging to four degrees of decay (DD 1–4) in Białowieża National Park (B) and Karkonosze National Park (K). **a** Oribatida (total Inertia 0.3946), **b** Mesostigmata (total Inertia 0.4420)

The differences between the samples belonging to particular DDs and parks were also indicated by PCoA (Fig. 9), separately for oribatid and mesostigmatid mites. The mite groups could be distinguished between the study sites. The permutational test of species selection, which is most strongly correlated with PCoA1 and PCoA2 (multiple regression), was conducted. The test allowed to indicate the species typical for each of the parks and DD for the two groups of mites separately. For Oribatida the typical species for BNP were (Fig. 9, Table 4): *Carabodes subarcticus*, *Damaeus* sp., *Chamobates cuspidatus*, and *Parachipteria punctata*. For KNP they were: *Carabodes areolatus*, *Chamobates borealis*, *Scheloribates pallidulus*, *Cepheus dentatus*, *Belba corynopus*, *Carabodes labyrinthicus*, and *Carabodes reticulatus*. Both *Carabodes coriaceus* and *Carabodes femoralis* occurred in great numbers in both study sites. For Mesostigmata the typical species for BNP were: *Dendrolaelaps pini*, *Trichouropoda ovalis*, *Sejus togatus*, and *Zercon curiosus*. And for KNP they were: *Zercon storkani*, *Zerconopsis remiger*, *Lasioseius zerconoides*, and *Dendrolaelaps cornutus*.

**Table 4:** Typical species names, the parks where they were found (BNP – Białowieża National Park, KNP – Karkonosze National Park), the ‘degree of decay’ they were associated with (DD 1–4; see Table 1 for explanation), and their abbreviations used in Fig. 9

Oribatida			Mesostigmata	
Names	Abbrev	Names		Abbrev
*Achipteria* sp. (BNP) *Belba corynopus* (DD 1) *Carabodes areolatus* (KNP) *Carabodes coriaceus* (DD 2) *Carabodes femoralis* (DD 2) *Carabodes labyrinthicus* (KNP) *Carabodes reticulatus* (KNP) *Carabodes subarcticus* (BNP) *Carabodes reticulatus* (DD 2) *Carabodes tenuis* (KNP) *Cepheus dentatus* (DD 1) *Chamobates borealis* (KNP) *Chamobates cuspidatus* (BNP) *Damaeus* sp. (BNP) *Dissorhina ornata* (DD 1) *Neoribates aurantiacus* (BNP) *Oppiella* (*Moritzoppia) unicarinata* (DD 1) *Oribatella calcarata* (KNP) *Parachipteria punctata* (BNP) *Scheloribates pallidulus* (KNP) *Steganacarus* (*Atropacarus*) *striculus* (DD 1)	Achisp Belbcory Caraareo Caracori Carafemo Caralaby Carareti Carasuba Carareti Caratenu Cephdent Chambore Camcusp Damasp Dissorna Neoraura Oppiunic Oribcalc Parapunc Schepall Stegstri	*Dendrolaelaps cornutus* (KNP) *Dendrolaelaps pini* (BNP) *Dinychus perforatus* (BNP) *Hoploseius oblongus* (DD 1) *Lasioseius zerconoides* (KNP) *Microgynium rectangulatum* (BNP) *Pergamasus rühmi* (KNP) *Sejus togatus* (BNP) *Trichouropoda ovalis* (BNP) *Zercon curiosus* (BNP) *Zercon storkani* (KNP) *Zercon remiger* (KNP)		Dendcorn Dendpini Dinyperf Hoploblo Lasizerc Micrrect Pergrühm Sejutoga Tricoval Zerccuri Zercstor Zercremi

## Discussion

Rarely studies were undertaken to indicate and define the factors, that influence the shape of invertebrate assemblages inhabiting the fruiting bodies of bracket fungi. Previous studies have been primarily faunistic, focusing on, e.g., insects (Jonsell and Nordlander 2004), spiders (Pielou and Pielou 1968) or mites (Gwiazdowicz and Łakomy 2002; Hågvar and Steen 2013).

### Mite assemblages

Anthropogenic pressure can have a strong and negative impact on the forest environment, which naturally also affects mite communities, e.g., soil Uropodina or Oribatida (Błoszyk 1999; Klimek and Rolbiecki 2014). The BNP is an example of an ecosystem with a unique natural structure (Faliński 1986), a large variety of habitats and microhabitats, which is reflected in species richness, also of mites (e.g., Olszanowski and Błoszyk 1998; Gwiazdowicz 2000). However, the forests of the Karkonosze Mountains have been marked by a strong human pressure (e.g., deformation of the structure of stands, degradation of the soil environment, toxic emissions). The current study demonstrates the differences between mite groupings in fruiting bodies of *F. pinicola* in forests similar to the natural forest of BNP and the ecosystems of KNP which were subjected to a strong anthropogenic impact. The parks differed in species composition. Despite a lower number of mite individuals, BNP samples were richer in species. Differences between the parks are apparent not only in terms of species presence or absence, but also in the proportions in which particular species occur – for example, *Carabodes subarcticus*, the third most numerous species in BNP, did not occur in KNP; or *Carabodes areolatus*, the second most numerous of the mites in KNP, was much less numerous than in BNP. Among the possible explanations of faunal differences among the parks could be the diverse specificity resulting from their lowland or mountain character, but also a different level of diversity of habitats and microhabitats, resulting from, e.g., human pressure.

### The influence of the degree of decay of fruiting bodies on mite assemblages

Our hypothesis that mite groupings would differ among fruiting bodies in various DDs was supported – the least decayed samples were characterised by lower species richness and mite individuals than the more decayed samples. The increase in the number of species and individuals with the degree of decay of fruiting bodies was non-linear. Particular mite species are characterized by different microhabitat preferences, the scale of tolerance and their requirements (Wehner et al. 2016). Some species occurred with a similar frequency regardless of the DD of fruiting bodies, but many species clearly preferred the fruiting bodies with a specific DD. As an example, *Hoploseius oblongus* was definitely the most abundant in the fruiting bodies of DD 1 in both parks, whereas *Carabodes femoralis* occurred most frequently in the more decayed samples (DD 3 and 4) in both study sites.

The differences between the mite fauna inhabiting the variously decayed fruiting bodies may result from differences in conditions (e.g., food availability) between the substrates. For instance, fresh fruiting bodies (DD 1) can create favourable conditions for presumably fungivorous *Hoploseius* mites (Lindquist 1965) or strongly decomposed fruiting bodies (DD 3 and 4) can be a substrate for saprotrophic fungi, which are part of the diet of such species as *Carabodes femoralis* (Schneider et al. 2005).

Our results can be compared to the results obtained in a similar microhabitat such as decaying wood. Similar to our results, Braccia and Batzer (2001), Skubała and Sokołowska (2006) and Gwiazdowicz et al. (2011) – looking at groupings in various phases of wood decay – found that with the greater decay of substrate, the species richness of inhabiting invertebrates usually increases. These authors attributed differences in the fauna to different characteristics of the substrate, e.g., moisture, density, or food resources, which can also be noted in fungal fruiting bodies.

### Species ecology

The mite species which occurred in the fruiting bodies of bracket fungi were also observed in other microhabitats, such as decaying wood or bark beetle galleries. However, among the mites appearing in the fruiting bodies of polypores a large percentage (up to 25%) of species inhabited this microhabitat exclusively (Salmane and Brumelis 2010). Consistent with the findings of Gdula et al. (2021), a significantly greater number of Oribatida (both species and individuals) than Mesostigmata was found. Also in other publications (e.g., Maraun et al. 2014), a significant share of oribatid mites in the invertebrate fauna of fungal fruiting bodies was shown, particularly of *Carabodes* spp. This genus is a dominant element of the fauna in other habitats as well, such as soil, bark of trees, and wood (Siira-Pietikäinen et al. 2008; Huhta et al. 2012; Hågvar and Steen 2013). *Carabodes* species are often common and numerous in similar habitats, such as soil and rotting wood (Schneider et al. 2005; Skubała and Sokołowska 2006), as well as on a wide range of consumable materials, such as the spores of many fungal species, or plant material (Maraun et al. 1998).

Mesostigmata are thought to use phoresy to colonize fruiting bodies (e.g., Błoszyk et al. 2006; Napierała and Błoszyk 2013). However, as these mites are mostly predators and occasional scavengers, some studies (Beaulieu 2012) suggest that some free-living predatory mites may be saproxylic, which may also be important in the case of microhabitat settlements similar to dead wood, such as bracket fungi. The most numerous species of Mesostigmata, *Dendrolaelaps pini*, was also found in fruiting bodies (Gdula et al. 2021), decayed wood (Gwiazdowicz et al. 2011), *Ips typographus* galleries (Salavatulin et al. 2018), in pine stumps and under the wing covers of *Hylurgus ligniperda* and *Hylastes* sp. (Hirschmann and Wiśniewski 1982). Another mesostigmatid mite species found in large numbers in these studies was *Hoploseius oblongus*, which had been previously described only in sporophores of *F. pinicola* in Slovakia (Mašán and Halliday 2016), and in fungal fruiting bodies in Poland (Gdula et al. 2021). *Zerconopsis remiger*, another abundant mesostigmatid mite found in sporocarps, was also extracted from soil (Manu et al. 2016; Karami et al. 2017), litter under *Populus* sp. (Salmane 2005), from starling nests (*Sturnus vulgaris*; Lesna et al. 2009), and in wood material, decaying organic material and from humus (Kalúz and Fenďa 2005). The most numerous mesostigmatid genus was *Dendrolaelaps*, represented by 12 species; they were also recorded in various habitats, such as Aphyllophorales fungi (e.g., *D*. *acornutus*, *D*. *cornutulus*, *D*. *punctatulus*), tree-hollows (*D*. *tenuipilus*, *D*. *zwoelferi*), or soil (*D*. *trapezoides*) (Salmane 2005; Salmane and Kontschan 2005; Kaczmarek et al. 2011).

In both studied locations, the most numerous mite species was *Carabodes femoralis*. This mycophagous species (Schneider et al. 2005) is known from sporocarps (Hågvar et al. 2014) and is also found in litter, which combines patchily distributed microhabitats that are inhabited by specialised species groups (Wehner et al. 2016) with soil (e.g., Błoszyk and Olszanowski 1997; Seniczak et al. 2006; Manu and Honciuc 2010), nests of *Formica rufa* ants (Sell 1990), cave mud, deadwood, leaves and guano (Maślak and Barczyk 2011). In addition, *Carabodes areolatus* – a species categorised as secondary decomposer (Nae et al. 2021) – was found not only in polypores (Hågvar and Steen 2013; Hågvar et al. 2014; Maraun et al. 2014), but also in soil (Błoszyk and Olszanowski 1997; Lebedeva and Krivolutsky 2003), feathers of hooded crow (*Corvus corone cornix*), magpie (*Pica pica*) and rook (*Corvus frugilegus*) (Krivolutsky and Lebedeva 2004). The third most numerous oribatid mite in the study – *Carabodes subarcticus* – was also found in such microhabitats as soil (e.g., Kagainis et al. 2010, 2015; Hågvar et al. 2014), touchwood, moss-covered branches (Żbikowska-Zdun et al. 2006) and bark of deadwood (Bluhm et al. 2015). The next species in terms of numbers was *Oribatella calcarata* which is ascribed to various trophic levels (Nae et al. 2021) and is known to inhabit such habitats as soil, cave mud, dead wood, leaves, and guano in the caves (Niedbała and Rohloff 1971; Maślak and Barczyk 2011).

Although some oribatid species reached high numbers, such as *C. femoralis* and *C*. *areolatus*, the vast majority were represented by only a few, or even a single individual and it could have been found in fruiting bodies by accident, passing by while moving along a tree trunk or in the litter. Some of the recorded Oribatida species, such as *Nothrus silvestris*, *Steganacarus* (*Steganacarus*) *magnus*, or *Damaeus* (*Paradamaeus*) *clavipes*, can be considered as fungivorous (Maraun et al. 2011), hence, they can use bracket fungi as a source of food. The hypotheses from Wehner et al. (2016), which were analysed in the context of oribatid mites occurring in the litter, may also be valuable for a better understanding of the formation of mite assemblages in fruiting bodies. Despite the differences between litter and fruiting bodies, some similarities potentially shape their fauna. Although oribatid mites are generally small, wingless and not highly dispersive, it has been shown that they can move using a variety of mechanisms, such as cursorial, active, or accidental transport and phoresy (Norton 1980; Krivolutsky and Lebedeva, 2004; Beaulieu et al. 2006; Ermilov and Frolov 2019), which enables them to successfully colonize the fruiting bodies of bracket fungi. Further research into the colonising mechanisms and the role of phoresy will help understand the diversity of mite assemblages in sporophores.

## Supplementary Information

Below is the link to the electronic supplementary material.

Supplementary file1 (DOCX 118 KB)

## Data Availability

Datasets are in the notebooks of authors. The specimens are deposited in acarological collections at the Department of Forest Pathology, Poznań University of Life Sciences, Poland (Mesostigmata) and the Institute of Biology, Biotechnology and Environmental Protection, University of Silesia, Poland (Oribatida).

## Code availability

No coded software was used for this study.

## References

[CR1] Bače R, Svoboda M, Pouska V, Janda P, Červenka J (2012). Natural regeneration in Central-European subalpine spruce forests: Which logs are suitable for seedling recruitment?. For Ecol Manag.

[CR2] Beaulieu F (2012). Saproxyly in predatory mites? Mesostigmata in decaying log habitats versus litter in a wet eucalypt forest, Tasmania. Australia Int J Acarol.

[CR3] Beaulieu F, Walter DE, Proctor HC, Kitching RL, Menzel F (2006). Mesostigmatid mites (Acari: Mesostigmata) on rainforest tree trunks: arboreal specialists, but substrate generalists?. Exp Appl Acarol.

[CR4] Błoszyk J (1999) Geograficzne i ekologiczne zróżnicowanie zrupowań roztoczy z kohorty Uropodina (Acari: Mesostigmata) w Polsce.I. Uropodina lasów grądowych. Wydawnictwo KONTEKST, Poznań

[CR5] Błoszyk J, Olszanowski Z (1997) Porównawcza analiza chorologiczna mechowców (Acari, Oribatida) Karkonoskiego i Gorczańskiego Parku Narodowego. Geoekologiczne Problemy Karkonoszy. Materiały z Sesji Naukowej w Przysiece 15–18 X 1997:109–114

[CR6] Błoszyk J, Klimczak J, Leśniewska M (2006). Phoretic relationships between Uropodina (Acari: Mesostigmata) and centipedes (Chilopoda) as an example of evolutionary adaptation of mites to temporary microhabitats. Eur J Entomol.

[CR7] Błoszyk J, Gutowski JM, Gwiazdowicz DJ, Mądra A, Konwerski S, Książkiewicz Z (2016). *Monochamus sartor* (Coleoptera: Cerambycidae) contributes to alpha diversity of Uropodina mites (Acari: Mesostigmata) in first stage of wood decay in Białowieża Primeval Forest. Int J Acarol.

[CR8] Bluhm C, Scheu S, Maraun M (2015). Oribatid mite communities on the bark of dead wood vary with log type, surrounding forest and regional factors. Appl Soil Ecol.

[CR9] Bowman JC, Sleep D, Forbes GJ, Edwards M (2000). The association of small mammals with coarse woody debris at log and stand scales. For Ecol Manag.

[CR10] Braccia A, Batzer DP (2001). Invertebrates associated with woody debris in a Southeastern US forested floodplain wetland. Wetlands.

[CR11] Butler R, Angelstam P, Ekelund P, Schlaepfer R (2004). Dead wood threshold values for the three-toed woodpecker presence in boreal and sub-Alpine forest. Biol Conserv.

[CR12] Cieśliński S, Porosty V, Okołów C, Karaś M, Bołbot A (2009). Białowieski Park Narodowy Poznać – Zrozumieć – Zachować.

[CR13] Conner RN, Miller OK, Adkisson CS (1976). Woodpecker dependence on trees infected by fungal heart rots. Wilson Bull.

[CR14] Dąbrowska-Prot E, Sarosiek J (1995). Problemy różnorodności biologicznej fauny w warunkach niszczenia borów świerkowych w Karkonoszach. Geoekologiczne Problemy Karkonoszy.

[CR15] Danielewicz W, Raj A, Zientarski J (2002) Ekosystemy leśne Karkonoskiego Parku Narodowego. Karkonoski Park Narodowy, Jelenia Góra

[CR16] Ehnström E (2001). Leaving dead wood for insects in boreal forests - suggestions for the future. Scand J for Res.

[CR17] Ermilov SG, Frolov AV (2019). *Ramusella* (*Dosangoppia*) *bochkovi* (Acari, Oribatida, Oppiidae), a new subgenus and species of oribatid mites phoretic on *Ceratophyus polyceros* (Pallas, 1771) (Coleoptera, Geotrupidae) from Russia. Syst Appl Acarol.

[CR18] Evans AM, Clinton PW, Allen RB, Frampton CM (2003). The influence of logs on the spatial distribution of litter-dwelling invertebrates and forest floor processes in New Zealand forests. For Ecol Manag.

[CR19] Faliński JB (1986). Vegetation dynamics in temperate forests (Ecological studies in Białowieża Forest).

[CR20] Gdula AK, Konwerski S, Olejniczak I, Rutkowski T, Skubała P, Zawieja B, Gwiazdowicz DJ (2021) The role of bracket fungi in creating alpha diversity of invertebrates in the Białowieża National Park. Poland Ecol Evol 11: 6456–6470. 10.1002/ece3.7495 10.1002/ece3.7495 PMC8207353 34141231

[CR21] Gutowski JM, Jaroszewicz B (2004). Białowieża Primeval Forest as a refuge of the European entomofauna. Wiad Entomol.

[CR22] Gwiazdowicz DJ (2000). Mites (Acari, Gamasida) of the Białowieża National Park. Sci Pap Agric Univ Poznań, Forestry.

[CR23] Gwiazdowicz DJ (2002). New species of *Hoploseius* Berlese 1914 (Acari: Gamasida, Ascidae) from Poland. Acta Zool Acad Sci Hung.

[CR24] Gwiazdowicz DJ (2003). Mites [Acari, Gamasida] of the tree stands in lower and upper subalpine forests in the Karkonosze National Park. Acta Sci Pol Silva Colend R Ind Lignaria.

[CR25] Gwiazdowicz DJ (2007) Ascid mites (Acari, Mesostigmata) from selected forest ecosystems and microhabitats in Poland. Wydawnictwo Akademii Rolniczej, Poznań

[CR26] Gwiazdowicz DJ (2010) Sejoidea, Antennophoroidea, Celaenopsoidea, Microgynioidea (Acari, Mesostigmata) of Poland. Bogucki Wydawnictwo Naukowe, Poznań

[CR27] Gwiazdowicz DJ, Biernacik R (2000). Roztocze (Acari, Gamasida) wybranych mikrośrodowisk Karkonoskiego Parku Narodowego. Opera Corcontica.

[CR28] Gwiazdowicz DJ, Kamczyc J, Rakowski R (2011) Mesostigmatid mites in four classes of wood decay. Exp Appl Acarol 55:155–165. 10.1007/s10493-011-9458-0 10.1007/s10493-011-9458-0 21479776

[CR29] Gwiazdowicz DJ, Łakomy P (2002). Mites (*Acari*, *Gamasida*) occurring in fruiting bodies of Aphyllophorales. Fragm Faun.

[CR30] Gwiazdowicz DJ, Gutowski JM, Kamczyc J, Teodorowicz E (2013). Phoretic relationships between *Plagionotus detritus* (Coleoptera: Cerambycidae) and *Trichouropoda sociata* (Acari: Mesostigmata). Entomol Fenn.

[CR31] Hågvar S, Steen R (2013). Succession of beetles (genus *Cis*) and oribatid mites (genus *Carabodes*) in dead sporocarps of the red-banded polypore fungus *Fomitopsis pinicola*. Scand J for Res.

[CR32] Hågvar S, Amundsen T, Økland B (2014). Mites of the genus *Carabodes* (Acari, Oribatida) in Norwegian coniferous forests: occurrence in different soils, vegetation types and polypore hosts. Scand J for Res.

[CR33] Hirschmann W, Wiśniewski J (1982) Weltweite Revision der Gattungen *Dendrolaelaps* Halbert 1915 und *Longoseius* Chant 1961 (Parasitiformes). Acarologie, Nürnberg

[CR34] Högberg N, Holdenrieder O, Stenlid J (1999). Population structure of the wood decay fungus *Fomitopsis pinicola*. Heredity.

[CR35] Huhta V, Siira-Pietikäinen A, Penttinen R (2012). Importance of dead wood for soil mite (Acarina) communities in boreal old-growth forests. Soil Org.

[CR36] Jadczyk P (2008) Przyrodnicze skutki wielkoobszarowego zamierania lasu w Sudetach Zachodnich. Wyd. IIiOŚ PW: 139–146

[CR37] Jaroszewicz B (2010) Charakterystyka przyrodnicza i historia Puszczy Białowieskiej i jej przedpola. In: Obidziński A Z Mazowszana Polesie i Wileńszczyznę. Zróżnicowanie i ochrona szaty roślinnej pogranicza Europy Środkowej i Północno-Wschodniej. Monograph of field sessions of the 55th Meeting of the Polish Botanical Society Planta in vivo, in vitro et in silico Warszawa, 6–12 September 2010

[CR38] Jędrzejewska B, Jędrzejewski W (1998) Predation in vertebrate communities. The Białowieża Primeval Forest as a case study. Springer, Berlin

[CR39] Jonsell M, Nordlander G (2004). Host selection patterns in insects breeding in bracket fungi. Ecol Entomol.

[CR40] Kaczmarek S, Marquardt T, Faleńczyk-Koziróg K (2011). Diversity of the Mesostigmata (Acari) in tree-hollows of selected deciduous tree species. Biol Lett.

[CR41] Kagainis U (2015). Use of quantitative morphological analysis combined with a large sample size for estimating morphological variability in a case study of Armoured Mite *Carabodes subarcticus* Trägårdh, 1902 (Acari: Oribatida: Carabodidae). Proc Latv Acad Sci B.

[CR42] Kagainis U (2010) *Carabodes rugosior* Berlese, *C. subarcticus* Trägardh 2010 – New Species of Oribatid Mites (Acari: Oribatida: Carabodidae) for Fauna of Latvia, with Brief Discussion of Their Microscoping. Latvijas Entomologs 2010(48): 115-117

[CR43] Kalúz S, Fenďa P (2005) Mites (Acari: Mesostigmata) of the family Ascidae of Slovakia. Institute of Zoology, Slovak Academy of Sciences, Bratislava

[CR44] Kappes H, Topp W (2004). Emergence of Coleoptera from deadwood in a managed broadleaved forest in central Europe. Biodivers Conserv.

[CR45] Karami F, Hajizadeh J, Ostovan H (2017). Fauna of Ascoidea (except Ameroseiidae) in Guilan Province, Iran with two new species record for Iran mites fauna. Linz Biol Beitr.

[CR46] Karg W (1993) Acari (Acarina), Milben, Parasitiformes (Anactinochaeta), Cohors Gamasina Leach. Raubmilben. Die Tierwelt Deutschlands, 59. Teil. Gustav Fischer Verlag, Jena

[CR47] Klimek A, Rolbiecki S (2014). Moss mites (Acari: Oribatida) in soil revitalizing: a chance for practical application in silviculture. Biol Lett.

[CR48] Kozłowski S, Żukowski K (1958). Investigations on Parasitic Gamasides in Białowieża National Park. Przegl Epidemiol.

[CR49] Krivolutsky DA, Lebedeva NV (2004). Oribatid mites (Oribatei) in bird feathers: Passeriformes. Acta Zool Litu.

[CR50] Kujawa A, Okołów C, Karaś M, Bołbot A (2009). VI Grzyby wielkoowocnikowe. Białowieski Park Narodowy Poznać – Zrozumieć – Zachować Białowieski Park.

[CR51] Kwiatkowski W, Gajko K (2009) Białowieski Park Narodowy, krajobrazy roślinne. In: Okołów C, Karaś M., & Bołbot A. Białowieski Park Narodowy. Poznać-Zrozumieć-Zachować III. Białowieża, BPN, pp 40–41

[CR52] Łakomy P, Kwaśna H (2008) Atlas hub. MULTICO Oficyna Wydawnicza, Warszawa

[CR53] Lebedeva NV, Krivolutsky DA (2003). Birds spread soil microarthropods to Arctic islands. Dokl Biol Sci.

[CR54] Lesna I, Wolfs P, Faraji F, Roy L, Komdeur J, Sabelis MW (2009). Candidate predators for biological control of the poultry red mite *Dermanyssus gallinae*. Exp Appl Acarol.

[CR55] Lindquist EE (1965). An Unusual New Species of *Hoploseius* Berlese (Acarina: Blattisociidae) from Mexico. Can Entomol.

[CR56] Lindquist EE (1995). Remarkable convergence between two taxa of ascid mites (Acari: Mesostigmata) adapted to living in pore tubes of bracket fungi in North America, with description of *Mycolaelaps* new genus. Can J Zool.

[CR57] Lonsdale D, Pautasso M (2008). Holdenrieder O (2007) Wood-decaying fungi in the forest: conservation needs and management options. Eur J for Res.

[CR58] Manu M, Honciuc V (2010). Ecological research on the soil mites populations (Acari: Mesostigmata-Gamasina, Oribatida) from forest ecosystems near Bucharest city. Rom J Biol - Zool.

[CR59] Manu M, Iordache V, Băncilă RI, Bodescu F, Onete M (2016). The influence of environmental variables on soil mite communities (Acari: Mesostigmata) from overgrazed grassland ecosystems – Romania. Ital J Zool.

[CR60] Maraun M, Migge S, Schaefer M, Scheu S (1998). Selection of microfungal food by six oribatid mite species (Oribatida, Acari) from two different beech forests. Pedobiologia.

[CR61] Maraun M, Erdmann G, Fischer BM, Pollierer MM, Norton RA, Schneider K, Scheu S (2011). Stable isotopes revisited: their use and limits for oribatid mite trophic ecology. Soil Biol Biochem.

[CR62] Maraun M, Augustin D, Müller J, Bässler C, Scheu S (2014). Changes in the community composition and trophic structure of microarthropods in sporocarps of the wood decaying fungus *Fomitopsis pinicola* along an altitudinal gradient. Appl Soil Ecol.

[CR63] Mašán P (2001). Mites of the cohort Uropodina (Acarina, Mesostigmata) in Slovakia. Annot Zool Bot.

[CR64] Mašán P, Halliday B (2016). A new species of *Hoploseius* (Acari: Blattisociidae) associated with the red-belted bracket fungus, *Fomitopsis pinicola* (Polyporaceae) in Slovakia. Syst Appl Acarol.

[CR65] Maser C, Trappe JM, Nussbaum RA (1978). Fungal—small mammal interrelationships with emphasis on Oregon coniferous forests. Ecology.

[CR66] Maślak M, Barczyk G (2011). Oribatid mites (Acari, Oribatida) in selected caves of the Kraków-Wieluń Upland (southern Poland). Biol Lett.

[CR67] McClelland BR, McClelland PT (1999). Pileated woodpecker nest and roost trees in Montana: links with old-growth and forest "health". Wildl Soc Bull.

[CR68] Nae I, Nae A, Scheu S, Maraun M (2021). Oribatid mite communities in mountain scree: stable isotopes (^15^N, ^13^C) reveal three trophic levels of exclusively sexual species. Exp Appl Acarol.

[CR69] Napierała A, Błoszyk J (2013). Unstable microhabitats (merocenoses) as specific habitats of Uropodina mites (Acari: Mesostigmata). Exp Appl Acarol.

[CR70] Niedbała W, Iwan D, Makol J (2008). Ptyctimous mites (Acari, Oribatida) of Poland. Fauna in Poloniae.

[CR71] Niedbała W, Rohloff J (1971). Wydajność aparatu Macfadyena w wypłaszaniu roztoczy glebowych. Roczniki Gleboznawcze.

[CR72] Niemelä T (2013) Polypores of the Białowieża Forest. Białowieski Park Narodowy, Białowieża

[CR73] Norton RA (1980). Observations on phoresy by oribatid mites (Acari: Oribatei). Int J Acarol.

[CR74] Norton RA (1990) Acarina: Oribatida. In: Dindal, D. L. (1990) Soil Biology Guide. J. Wiley & Sons, New York, pp 779–803

[CR75] O'Connell T, Bolger T (1997). Fungal fruiting bodies and the structure of fungus-micro-arthropod assemblages. Biol Environ: Proc Roy Irish Acad.

[CR76] O'Connell T, Bolger T (1997). Stability, ephemerality and dispersal ability: microarthropod assemblages on fungal sporophores. Biol J Linn Soc.

[CR77] Oksanen J, Blanchet FG, Friendly M, Kindt R, Legendre P, McGlinn D, Minchin PR, O'Hara RB, Simpson GL, Solymos P, Stevens MHH, Szoecs E, Wagner H (2019) *vegan: Community Ecology Package.* R package version 2.5–6.2019. https://CRAN.R-project.org/package=vegan

[CR78] Olszanowski Z (1996) A Monograph of the Nothridae and Camisiidae of Poland (Acari: Oribatida: Crotonioidea). Genus (Supplement), Wrocław

[CR79] Olszanowski Z, Błoszyk J (1998). Materiały do znajomości akarofauny Puszczy Białowieskiej I Mechowce (Acari: Oribatida). Parki Narodowe i Rezerwaty Przyrody.

[CR80] Pielou DP, Pielou EC (1968). Association among species of infrequent occurrence: the insect and spider fauna of *Polyporus betulinus* (Bulliard) Fries. J Theor Biol.

[CR81] Pielou DP, Verma AN (1968). The arthropod fauna associated with the birch bracket fungus, *Polyporus betulinus*, in eastern Canada. Can Entomol.

[CR82] Porter AD, St Clair CC, Vries AD (2005). Fine-scale selection by marten during winter in a young deciduous forest. Can J for Res.

[CR83] Raj A, Knapik R (2014) Karkonoski Park Narodowy II Wydanie. Karkonoski Park Narodowy, Jelenia Góra

[CR84] Rajski A (1961). Studium ekologiczno-faunistyczne nad mechowcami (Acari, Oribatei) w kilku zespołach roślinnych. Pr Kom Mat Przyr PTPN.

[CR85] Salavatulin VM, Trach VA, Bobylev AN, Khaustov VA, Tolstikov AV, Khaustov AA, Klimov PB (2018). Review of Mites (Acari) Associated with the European Spruce Bark Beetle, *Ips typographus* (Coleoptera: Curculionidae: Scolytinae) in Asian Russia. Acarina.

[CR86] Salmane I (2005). Addition to the Latvian Mesostigmata (Acari, Parasitiformes) check-list. Latvijas Entomologs.

[CR87] Salmane I, Brumelis G (2010). Species list and habitat preference of Mesostigmata mites (Acari, Parasitiformes) in Latvia. Acarologia.

[CR88] Salmane I, Kontschan J (2005). Soil Gamasina mites (Acari, Parasitiformes, Mesostigmata) from Hungary. Latvijas Entomologs.

[CR89] Schneider K, Renker C, Maraun M (2005). Oribatid mite (Acari, Oribatida) feeding on ectomycorrhizal fungi. Mycorrhiza.

[CR90] Sell D (1990) Mechowce (Acari, Oribatei) z gniazd mrówek *Formica rufa* (S.L) z terenów Bieszczadzkiego Parku Narodowego. Zesz Probl Postęp Nauk Rol 373:151–161

[CR91] Seniczak S, Bukowski G, Seniczak A (2006) Mechowce (Acari, Oribatida) glebowe strefy ekotonowej pomiędzy borem sosnowym a brzegiem jeziora lobeliowego Małe Gacno w Borach Tucholskich Uniwersytet Technologiczno-Przyrodniczy im Jana i Jędrzeja Śniadeckich w Bydgoszczy Zeszyty Naukowe. Zootechnika Uniwersytet Technologiczno-Przyrodniczy w Bydgoszczy 36: 45–50

[CR92] Shah F, Mali T, Lundell TK (2018). Polyporales brown rot species *Fomitopsis pinicola*: Enzyme activity profiles, oxalic acid production, and Fe3+-reducing metabolite secretion. Appl Environ Microbiol.

[CR93] Siira-Pietikäinen A, Penttinen R, Huhta V (2008). Oribatid mites (Acari: Oribatida) in boreal forest floor and decaying wood. Pedobiologia.

[CR94] Skubała P, Duras M (2008). Do decaying logs represent habitat islands? Oribatid mite communities in dead wood. Ann Zool.

[CR95] Skubała P, Sokołowska M (2006). Oribatid fauna (Acari, Oribatida) in fallen spruce trees in the Babia Góra National Park. Biol Lett.

[CR96] Sobik M, Błaś M (2008) Natural and human impact on pollutant deposition in mountain ecosystems with the Sudetes as an example. In EE’08 Proceedings of the 3rd IASME/WSEAS International Conference on Energy & Environment. Cambridge, pp 355-359

[CR97] Sokołowski AW (2004) Lasy Puszczy Białowieskiej. Centrum Informacyjne Lasów Państwowych, Bedoń

[CR98] Suter W, Schielly B (1998). Liegendes Totholz: Ein wichtiges Strukturmerkmal für die Habitatqualität von Kleinsäugern und kleinen Carnivoren im Wald. Schweiz Z Forstwes.

[CR99] Szymura TH, Dunajski A, Ruczakowska AM (2010). Zmiany powierzchni lasów na obszarze Karkonoskiego Parku Narodowego w okresie 1747–1977. Opera Corcon.

[CR100] Thunes KH, Willassen E (1997). Species composition of beetles (Coleoptera) in the bracket fungi *Piptoporus betulinus* and *Fomes fomentarius* (Aphyllophorales: Polyporaceae): an explorative approach with canonical correspondence analysis. J Nat Hist.

[CR101] Tomiałojć L (1991). Characteristics of old growth in the Bialowieza Forest. Poland Nat Areas J.

[CR102] Walter DE (1998). *Hoploseius australianus* sp. nov (Acari: Mesostigmata: Ascidae), a unique element in the Australian acarofauna. Aust Entomol.

[CR103] Wehner K, Norton RA, Blüthgen N, Heethoff M (2016). Specialization of oribatid mites to forest microhabitats — the enigmatic role of litter. Ecosphere.

[CR104] Weigmann G, Dahl F (2006). Hornmilben (Oribatida). Die Tierwelt Deutschlands und der angrenzenden Meeresteile, 76, Teil.

[CR106] Żbikowska-Zdun K, Piksa K, Watrak I (2006). Diversity of mites (Acari: Oribatida) in selected microhabitats of the bug river protected landscape area. Biol Lett.

