# Supplementary material for: Mite communities (Acari: Mesostigmata, Oribatida) in the red belt conk, Fomitopsis pinicola (Polyporales), in Polish forests
Source: Exp Appl Acarol. 2021 Jun 29;84(3):543–64. doi: 10.1007/s10493-021-00635-1 (PMC8257520; doi:10.1007/s10493-021-00635-1)
Supplement: Supplementary file 1 — Supplementary file1 (DOCX 118 KB) [file 10493_2021_635_MOESM1_ESM.docx]

|  | **BNP 1 DD** | | | **BNP 2 DD** | | | **BNP 3 DD** | | | **BNP 4 DD** | | | **BNP Total** | | |
| --- | --- | --- | --- | --- | --- | --- | --- | --- | --- | --- | --- | --- | --- | --- | --- |
| Species | Abu | Fre | Dom | Abu | Fre | Dom | Abu | Fre | Dom | Abu | Fre | Dom | Abu | Fre | Dom |
| **Acari, Mesostigmata** | 85 | 1.00 | 0.54 | 14 | 0.83 | 0.25 | 113 | 0.91 | 0.11 | 612 | 1.00 | 0.20 | 824 | 0.95 | 0.19 |
| *Amblyseius* sp. | 0 | 0.00 | 0.00 | 0 | 0.00 | 0.00 | 0 | 0.00 | 0.00 | 1 | 0.05 | 0.00 | 1 | 0.03 | 0.00 |
| *Dendrolaelaps acornutus* | 0 | 0.00 | 0.00 | 0 | 0.00 | 0.00 | 12 | 0.18 | 0.01 | 18 | 0.05 | 0.01 | 30 | 0.08 | 0.01 |
| *Dendrolaelaps arvicolis* | 1 | 0.25 | 0.01 | 0 | 0.00 | 0.00 | 0 | 0.00 | 0.00 | 0 | 0.00 | 0.00 | 1 | 0.03 | 0.00 |
| *Dendrolaelaps cornutulus* | 2 | 0.25 | 0.01 | 0 | 0.00 | 0.00 | 0 | 0.00 | 0.00 | 0 | 0.00 | 0.00 | 2 | 0.03 | 0.00 |
| *Dendrolaelaps euarmatus* | 0 | 0.00 | 0.00 | 0 | 0.00 | 0.00 | 0 | 0.00 | 0.00 | 1 | 0.05 | 0.00 | 1 | 0.03 | 0.00 |
| *Dendrolaelaps pini* | 0 | 0.00 | 0.00 | 0 | 0.00 | 0.00 | 31 | 0.64 | 0.03 | 237 | 0.53 | 0.08 | 268 | 0.43 | 0.06 |
| *Dendrolaelaps procornutus* | 2 | 0.25 | 0.01 | 0 | 0.00 | 0.00 | 0 | 0.00 | 0.00 | 0 | 0.00 | 0.00 | 2 | 0.03 | 0.00 |
| *Dendrolaelaps punctatulus* | 0 | 0.00 | 0.00 | 0 | 0.00 | 0.00 | 0 | 0.00 | 0.00 | 36 | 0.11 | 0.01 | 36 | 0.05 | 0.01 |
| *Dendrolaelaps* sp. | 0 | 0.00 | 0.00 | 0 | 0.00 | 0.00 | 0 | 0.00 | 0.00 | 2 | 0.11 | 0.00 | 2 | 0.05 | 0.00 |
| *Dendrolaelaps tenuipilus* | 0 | 0.00 | 0.00 | 0 | 0.00 | 0.00 | 0 | 0.00 | 0.00 | 4 | 0.05 | 0.00 | 4 | 0.03 | 0.00 |
| *Dendrolaelaps trapezoides* | 0 | 0.00 | 0.00 | 0 | 0.00 | 0.00 | 3 | 0.18 | 0.00 | 0 | 0.00 | 0.00 | 3 | 0.05 | 0.00 |
| *Dendrolaelaps zwoelferi* | 0 | 0.00 | 0.00 | 1 | 0.17 | 0.02 | 13 | 0.09 | 0.01 | 12 | 0.11 | 0.00 | 26 | 0.10 | 0.01 |
| *Dinychus arcuatus* | 0 | 0.00 | 0.00 | 0 | 0.00 | 0.00 | 0 | 0.00 | 0.00 | 15 | 0.26 | 0.00 | 15 | 0.13 | 0.00 |
| *Dinychus perforatus* | 0 | 0.00 | 0.00 | 0 | 0.00 | 0.00 | 0 | 0.00 | 0.00 | 83 | 0.37 | 0.03 | 83 | 0.18 | 0.02 |
| *Gamasellodes bicolor* | 0 | 0.00 | 0.00 | 0 | 0.00 | 0.00 | 2 | 0.09 | 0.00 | 0 | 0.00 | 0.00 | 2 | 0.03 | 0.00 |
| *Gamasellus montanus* | 0 | 0.00 | 0.00 | 0 | 0.00 | 0.00 | 0 | 0.00 | 0.00 | 1 | 0.05 | 0.00 | 1 | 0.03 | 0.00 |
| *Geholaspis longispinosus* | 0 | 0.00 | 0.00 | 0 | 0.00 | 0.00 | 0 | 0.00 | 0.00 | 1 | 0.05 | 0.00 | 1 | 0.03 | 0.00 |
| *Geholaspis mandibularis* | 0 | 0.00 | 0.00 | 0 | 0.00 | 0.00 | 0 | 0.00 | 0.00 | 1 | 0.05 | 0.00 | 1 | 0.03 | 0.00 |
| *Hoploseius oblongus* | 79 | 1.00 | 0.50 | 8 | 0.67 | 0.15 | 0 | 0.00 | 0.00 | 1 | 0.05 | 0.00 | 88 | 0.23 | 0.02 |
| *Pneumolaelaps lubrica* | 0 | 0.00 | 0.00 | 0 | 0.00 | 0.00 | 0 | 0.00 | 0.00 | 4 | 0.11 | 0.00 | 4 | 0.05 | 0.00 |
| *Iphidonopsis dendrophilus* | 0 | 0.00 | 0.00 | 0 | 0.00 | 0.00 | 2 | 0.09 | 0.00 | 0 | 0.00 | 0.00 | 2 | 0.03 | 0.00 |
| *Lasioseius fimetorum* | 0 | 0.00 | 0.00 | 0 | 0.00 | 0.00 | 0 | 0.00 | 0.00 | 3 | 0.05 | 0.00 | 3 | 0.03 | 0.00 |
| *Lasioseius ometes* | 0 | 0.00 | 0.00 | 1 | 0.17 | 0.02 | 0 | 0.00 | 0.00 | 61 | 0.21 | 0.02 | 62 | 0.13 | 0.01 |
| *Microgynium rectangulatum* | 0 | 0.00 | 0.00 | 0 | 0.00 | 0.00 | 3 | 0.09 | 0.00 | 5 | 0.26 | 0.00 | 8 | 0.15 | 0.00 |
| *Parazercon radiatus* | 0 | 0.00 | 0.00 | 0 | 0.00 | 0.00 | 1 | 0.09 | 0.00 | 0 | 0.00 | 0.00 | 1 | 0.03 | 0.00 |
| *Pergamasus* sp. | 0 | 0.00 | 0.00 | 0 | 0.00 | 0.00 | 0 | 0.00 | 0.00 | 1 | 0.05 | 0.00 | 1 | 0.03 | 0.00 |
| *Pleuronectocelaeno austriaca* | 0 | 0.00 | 0.00 | 0 | 0.00 | 0.00 | 0 | 0.00 | 0.00 | 2 | 0.11 | 0.00 | 2 | 0.05 | 0.00 |
| *Proctolaelaps pygmaeus* | 0 | 0.00 | 0.00 | 0 | 0.00 | 0.00 | 0 | 0.00 | 0.00 | 1 | 0.05 | 0.00 | 1 | 0.03 | 0.00 |
| *Sejus togatus* | 0 | 0.00 | 0.00 | 0 | 0.00 | 0.00 | 9 | 0.18 | 0.01 | 31 | 0.26 | 0.01 | 40 | 0.18 | 0.01 |
| *Trichouropoda ovalis* | 0 | 0.00 | 0.00 | 1 | 0.17 | 0.02 | 28 | 0.27 | 0.03 | 18 | 0.37 | 0.01 | 47 | 0.28 | 0.01 |
| *Uroobovella vinicolora* | 0 | 0.00 | 0.00 | 0 | 0.00 | 0.00 | 7 | 0.18 | 0.01 | 0 | 0.00 | 0.00 | 7 | 0.05 | 0.00 |
| *Veigaia kochi* | 0 | 0.00 | 0.00 | 0 | 0.00 | 0.00 | 0 | 0.00 | 0.00 | 2 | 0.11 | 0.00 | 2 | 0.05 | 0.00 |
| *Veigaia nemorensis* | 0 | 0.00 | 0.00 | 0 | 0.00 | 0.00 | 0 | 0.00 | 0.00 | 13 | 0.32 | 0.00 | 13 | 0.15 | 0.00 |
| *Veigaia transisale* | 0 | 0.00 | 0.00 | 0 | 0.00 | 0.00 | 2 | 0.09 | 0.00 | 5 | 0.16 | 0.00 | 7 | 0.10 | 0.00 |
| *Zercon curiosus* | 1 | 0.25 | 0.01 | 3 | 0.33 | 0.05 | 0 | 0.00 | 0.00 | 50 | 0.47 | 0.02 | 54 | 0.30 | 0.01 |
| *Zerconopsis decemremiger* | 0 | 0.00 | 0.00 | 0 | 0.00 | 0.00 | 0 | 0.00 | 0.00 | 1 | 0.05 | 0.00 | 1 | 0.03 | 0.00 |
| *Zerconopsis remiger* | 0 | 0.00 | 0.00 | 0 | 0.00 | 0.00 | 0 | 0.00 | 0.00 | 2 | 0.05 | 0.00 | 2 | 0.03 | 0.00 |
| **Acari, Oribatida** | 73 | 0.75 | 0.46 | 41 | 1.00 | 0.75 | 943 | 0.91 | 0.89 | 2464 | 0.89 | 0.80 | 3521 | 0.90 | 0.81 |
| *Achipteria coleoptrata* | 0 | 0.00 | 0.00 | 0 | 0.00 | 0.00 | 0 | 0.00 | 0.00 | 9 | 0.11 | 0.00 | 9 | 0.05 | 0.00 |
| *Achipteria nitens* | 0 | 0.00 | 0.00 | 0 | 0.00 | 0.00 | 0 | 0.00 | 0.00 | 24 | 0.11 | 0.01 | 24 | 0.05 | 0.01 |
| *Achipteria* sp. | 0 | 0.00 | 0.00 | 0 | 0.00 | 0.00 | 0 | 0.00 | 0.00 | 28 | 0.21 | 0.01 | 28 | 0.10 | 0.01 |
| *Acrogalumna longipluma* | 0 | 0.00 | 0.00 | 0 | 0.00 | 0.00 | 2 | 0.09 | 0.00 | 2 | 0.11 | 0.00 | 4 | 0.08 | 0.00 |
| *Autogneta longilamellata* | 8 | 0.25 | 0.05 | 0 | 0.00 | 0.00 | 0 | 0.00 | 0.00 | 6 | 0.05 | 0.00 | 14 | 0.05 | 0.00 |
| *Caleremaeus monilipes* | 1 | 0.25 | 0.01 | 0 | 0.00 | 0.00 | 0 | 0.00 | 0.00 | 1 | 0.05 | 0.00 | 2 | 0.05 | 0.00 |
| *Carabodes areolatus* | 0 | 0.00 | 0.00 | 1 | 0.17 | 0.02 | 0 | 0.00 | 0.00 | 7 | 0.21 | 0.00 | 8 | 0.13 | 0.00 |
| *Carabodes coriaceus* | 0 | 0.00 | 0.00 | 1 | 0.17 | 0.02 | 13 | 0.27 | 0.01 | 8 | 0.11 | 0.00 | 22 | 0.15 | 0.01 |
| *Carabodes femoralis* | 14 | 0.75 | 0.09 | 20 | 0.83 | 0.36 | 771 | 0.73 | 0.73 | 1961 | 0.79 | 0.64 | 2766 | 0.78 | 0.64 |
| *Carabodes labyrinthicus* | 0 | 0.00 | 0.00 | 2 | 0.33 | 0.04 | 0 | 0.00 | 0.00 | 5 | 0.16 | 0.00 | 7 | 0.13 | 0.00 |
| *Carabodes ornatus* | 0 | 0.00 | 0.00 | 0 | 0.00 | 0.00 | 4 | 0.09 | 0.00 | 9 | 0.26 | 0.00 | 13 | 0.15 | 0.00 |
| *Carabodes subarcticus* | 16 | 0.75 | 0.10 | 5 | 0.50 | 0.09 | 99 | 0.64 | 0.09 | 141 | 0.74 | 0.05 | 261 | 0.68 | 0.06 |
| *Carabodes* sp. | 2 | 0.25 | 0.01 | 0 | 0.00 | 0.00 | 0 | 0.00 | 0.00 | 1 | 0.05 | 0.00 | 3 | 0.05 | 0.00 |
| *Carabodes tenuis* | 1 | 0.25 | 0.01 | 0 | 0.00 | 0.00 | 0 | 0.00 | 0.00 | 0 | 0.00 | 0.00 | 1 | 0.03 | 0.00 |
| *Cepheus cepheiformis* | 0 | 0.00 | 0.00 | 0 | 0.00 | 0.00 | 3 | 0.18 | 0.00 | 18 | 0.21 | 0.01 | 21 | 0.15 | 0.00 |
| *Cepheus* sp. | 0 | 0.00 | 0.00 | 0 | 0.00 | 0.00 | 0 | 0.00 | 0.00 | 1 | 0.05 | 0.00 | 1 | 0.03 | 0.00 |
| *Chamobates cuspidatus* | 4 | 0.50 | 0.03 | 2 | 0.17 | 0.04 | 1 | 0.09 | 0.00 | 12 | 0.26 | 0.00 | 19 | 0.23 | 0.00 |
| *Chamobates pusillus* | 1 | 0.25 | 0.01 | 0 | 0.00 | 0.00 | 0 | 0.00 | 0.00 | 0 | 0.00 | 0.00 | 1 | 0.03 | 0.00 |
| *Chamobates spinosus* | 1 | 0.25 | 0.01 | 0 | 0.00 | 0.00 | 0 | 0.00 | 0.00 | 0 | 0.00 | 0.00 | 1 | 0.03 | 0.00 |
| *Chamobates voigtsi* | 0 | 0.00 | 0.00 | 0 | 0.00 | 0.00 | 0 | 0.00 | 0.00 | 1 | 0.05 | 0.00 | 1 | 0.03 | 0.00 |
| *Cymbaeremaeus cymba* | 0 | 0.00 | 0.00 | 0 | 0.00 | 0.00 | 1 | 0.09 | 0.00 | 1 | 0.05 | 0.00 | 2 | 0.05 | 0.00 |
| *Damaeus* (*Adamaeus*) *onustus* | 0 | 0.00 | 0.00 | 0 | 0.00 | 0.00 | 0 | 0.00 | 0.00 | 3 | 0.11 | 0.00 | 3 | 0.05 | 0.00 |
| *Damaeus riparius* | 0 | 0.00 | 0.00 | 0 | 0.00 | 0.00 | 1 | 0.09 | 0.00 | 2 | 0.11 | 0.00 | 3 | 0.08 | 0.00 |
| *Damaeus* sp. | 1 | 0.25 | 0.01 | 5 | 0.50 | 0.09 | 3 | 0.09 | 0.00 | 11 | 0.26 | 0.00 | 20 | 0.25 | 0.00 |
| *Dissorhina ornata* | 1 | 0.25 | 0.01 | 0 | 0.00 | 0.00 | 0 | 0.00 | 0.00 | 4 | 0.11 | 0.00 | 5 | 0.08 | 0.00 |
| *Epidamaeus bituberculatus* | 0 | 0.00 | 0.00 | 0 | 0.00 | 0.00 | 0 | 0.00 | 0.00 | 4 | 0.11 | 0.00 | 4 | 0.05 | 0.00 |
| *Eniochthonius minutissimus* | 1 | 0.25 | 0.01 | 0 | 0.00 | 0.00 | 0 | 0.00 | 0.00 | 1 | 0.05 | 0.00 | 2 | 0.05 | 0.00 |
| *Epidamaeus setiger* | 1 | 0.25 | 0.01 | 0 | 0.00 | 0.00 | 0 | 0.00 | 0.00 | 0 | 0.00 | 0.00 | 1 | 0.03 | 0.00 |
| *Euphthiracarus cribrarius* | 0 | 0.00 | 0.00 | 0 | 0.00 | 0.00 | 1 | 0.09 | 0.00 | 1 | 0.05 | 0.00 | 2 | 0.05 | 0.00 |
| *Euzetes globulus* | 0 | 0.00 | 0.00 | 0 | 0.00 | 0.00 | 0 | 0.00 | 0.00 | 1 | 0.05 | 0.00 | 1 | 0.03 | 0.00 |
| *Fuscozetes setosus* | 0 | 0.00 | 0.00 | 0 | 0.00 | 0.00 | 2 | 0.09 | 0.00 | 0 | 0.00 | 0.00 | 2 | 0.03 | 0.00 |
| *Globozetes longipilus* | 0 | 0.00 | 0.00 | 0 | 0.00 | 0.00 | 0 | 0.00 | 0.00 | 4 | 0.11 | 0.00 | 4 | 0.05 | 0.00 |
| *Hafenrefferia gilvipes* | 0 | 0.00 | 0.00 | 0 | 0.00 | 0.00 | 0 | 0.00 | 0.00 | 11 | 0.16 | 0.00 | 11 | 0.08 | 0.00 |
| *Hypochthonius rufulus* | 0 | 0.00 | 0.00 | 0 | 0.00 | 0.00 | 0 | 0.00 | 0.00 | 28 | 0.05 | 0.01 | 28 | 0.03 | 0.01 |
| *Kunstidamaeus tecticola* | 0 | 0.00 | 0.00 | 0 | 0.00 | 0.00 | 0 | 0.00 | 0.00 | 1 | 0.05 | 0.00 | 1 | 0.03 | 0.00 |
| *Lagenobates lagenulus* | 2 | 0.50 | 0.01 | 0 | 0.00 | 0.00 | 0 | 0.00 | 0.00 | 2 | 0.05 | 0.00 | 4 | 0.08 | 0.00 |
| *Liacarus coracinus* | 1 | 0.25 | 0.01 | 0 | 0.00 | 0.00 | 1 | 0.09 | 0.00 | 1 | 0.05 | 0.00 | 3 | 0.08 | 0.00 |
| *Licneremaeus licnophorus* | 0 | 0.00 | 0.00 | 0 | 0.00 | 0.00 | 0 | 0.00 | 0.00 | 1 | 0.05 | 0.00 | 1 | 0.03 | 0.00 |
| *Liebstadia longior* | 0 | 0.00 | 0.00 | 1 | 0.17 | 0.02 | 0 | 0.00 | 0.00 | 0 | 0.00 | 0.00 | 1 | 0.03 | 0.00 |
| *Malaconothrus monodactylus* | 0 | 0.00 | 0.00 | 0 | 0.00 | 0.00 | 0 | 0.00 | 0.00 | 2 | 0.05 | 0.00 | 2 | 0.03 | 0.00 |
| *Melanozetes mollicomus* | 0 | 0.00 | 0.00 | 0 | 0.00 | 0.00 | 0 | 0.00 | 0.00 | 2 | 0.11 | 0.00 | 2 | 0.05 | 0.00 |
| *Metabelba* sp. | 0 | 0.00 | 0.00 | 0 | 0.00 | 0.00 | 0 | 0.00 | 0.00 | 1 | 0.05 | 0.00 | 1 | 0.03 | 0.00 |
| *Nanhermannia dorsalis* | 0 | 0.00 | 0.00 | 0 | 0.00 | 0.00 | 0 | 0.00 | 0.00 | 1 | 0.05 | 0.00 | 1 | 0.03 | 0.00 |
| *Nanhermannia nana* | 0 | 0.00 | 0.00 | 0 | 0.00 | 0.00 | 0 | 0.00 | 0.00 | 25 | 0.16 | 0.01 | 25 | 0.08 | 0.01 |
| *Neoliodes theleproctus* | 0 | 0.00 | 0.00 | 0 | 0.00 | 0.00 | 1 | 0.09 | 0.00 | 0 | 0.00 | 0.00 | 1 | 0.03 | 0.00 |
| *Neoribates aurantiacus* | 0 | 0.00 | 0.00 | 0 | 0.00 | 0.00 | 29 | 0.27 | 0.03 | 3 | 0.11 | 0.00 | 32 | 0.13 | 0.01 |
| *Nothrus silvestris* | 0 | 0.00 | 0.00 | 0 | 0.00 | 0.00 | 0 | 0.00 | 0.00 | 4 | 0.11 | 0.00 | 4 | 0.05 | 0.00 |
| *Oppia nitens* | 0 | 0.00 | 0.00 | 0 | 0.00 | 0.00 | 0 | 0.00 | 0.00 | 2 | 0.05 | 0.00 | 2 | 0.03 | 0.00 |
| *Oppiella* (*Moritzoppia*) *keilbachi* | 0 | 0.00 | 0.00 | 0 | 0.00 | 0.00 | 0 | 0.00 | 0.00 | 5 | 0.16 | 0.00 | 5 | 0.08 | 0.00 |
| *Oppiella* (*Moritzoppia*) *unicarinata* | 0 | 0.00 | 0.00 | 0 | 0.00 | 0.00 | 0 | 0.00 | 0.00 | 10 | 0.11 | 0.00 | 10 | 0.05 | 0.00 |
| *Oppiella* (*Oppiella*) *falcata* | 0 | 0.00 | 0.00 | 0 | 0.00 | 0.00 | 0 | 0.00 | 0.00 | 1 | 0.05 | 0.00 | 1 | 0.03 | 0.00 |
| *Oppiella* (*Oppiella*) *nova* | 1 | 0.25 | 0.01 | 0 | 0.00 | 0.00 | 0 | 0.00 | 0.00 | 4 | 0.05 | 0.00 | 5 | 0.05 | 0.00 |
| *Oppiella* (*Rhinoppia*) *subpectinata* | 3 | 0.25 | 0.02 | 0 | 0.00 | 0.00 | 0 | 0.00 | 0.00 | 1 | 0.05 | 0.00 | 4 | 0.05 | 0.00 |
| *Oppiella* sp. | 0 | 0.00 | 0.00 | 0 | 0.00 | 0.00 | 0 | 0.00 | 0.00 | 1 | 0.05 | 0.00 | 1 | 0.03 | 0.00 |
| *Oribatella calcarata* | 1 | 0.25 | 0.01 | 0 | 0.00 | 0.00 | 2 | 0.09 | 0.00 | 7 | 0.26 | 0.00 | 10 | 0.18 | 0.00 |
| *Oribatella sexdentata* | 0 | 0.00 | 0.00 | 0 | 0.00 | 0.00 | 1 | 0.09 | 0.00 | 3 | 0.11 | 0.00 | 4 | 0.08 | 0.00 |
| *Oribatella similesuperbula* | 0 | 0.00 | 0.00 | 0 | 0.00 | 0.00 | 0 | 0.00 | 0.00 | 2 | 0.05 | 0.00 | 2 | 0.03 | 0.00 |
| other juveniles | 0 | 0.00 | 0.00 | 1 | 0.17 | 0.02 | 0 | 0.00 | 0.00 | 10 | 0.21 | 0.00 | 11 | 0.13 | 0.00 |
| *Parachipteria punctata* | 9 | 0.50 | 0.06 | 0 | 0.00 | 0.00 | 0 | 0.00 | 0.00 | 14 | 0.11 | 0.00 | 23 | 0.10 | 0.01 |
| *Pergalumna nervosa* | 0 | 0.00 | 0.00 | 0 | 0.00 | 0.00 | 0 | 0.00 | 0.00 | 3 | 0.11 | 0.00 | 3 | 0.05 | 0.00 |
| *Phauloppia nemoralis* | 0 | 0.00 | 0.00 | 0 | 0.00 | 0.00 | 0 | 0.00 | 0.00 | 2 | 0.11 | 0.00 | 2 | 0.05 | 0.00 |
| *Phthiracarus anonymus* | 0 | 0.00 | 0.00 | 1 | 0.17 | 0.02 | 0 | 0.00 | 0.00 | 1 | 0.05 | 0.00 | 2 | 0.05 | 0.00 |
| *Phthiracarus bryobius* | 0 | 0.00 | 0.00 | 0 | 0.00 | 0.00 | 0 | 0.00 | 0.00 | 2 | 0.11 | 0.00 | 2 | 0.05 | 0.00 |
| *Phthiracarus compressus* | 0 | 0.00 | 0.00 | 2 | 0.17 | 0.04 | 0 | 0.00 | 0.00 | 3 | 0.05 | 0.00 | 5 | 0.05 | 0.00 |
| *Phthiracarus ferrugineus* | 0 | 0.00 | 0.00 | 0 | 0.00 | 0.00 | 0 | 0.00 | 0.00 | 2 | 0.05 | 0.00 | 2 | 0.03 | 0.00 |
| *Phthiracarus globosus* | 1 | 0.25 | 0.01 | 0 | 0.00 | 0.00 | 0 | 0.00 | 0.00 | 0 | 0.00 | 0.00 | 1 | 0.03 | 0.00 |
| *Phthiracarus longulus* | 1 | 0.25 | 0.01 | 0 | 0.00 | 0.00 | 2 | 0.18 | 0.00 | 8 | 0.16 | 0.00 | 11 | 0.15 | 0.00 |
| *Platynothrus peltifer* | 0 | 0.00 | 0.00 | 0 | 0.00 | 0.00 | 0 | 0.00 | 0.00 | 2 | 0.11 | 0.00 | 2 | 0.05 | 0.00 |
| *Platyliodes scaliger* | 0 | 0.00 | 0.00 | 0 | 0.00 | 0.00 | 0 | 0.00 | 0.00 | 1 | 0.05 | 0.00 | 1 | 0.03 | 0.00 |
| *Porobelba spinosa* | 0 | 0.00 | 0.00 | 0 | 0.00 | 0.00 | 0 | 0.00 | 0.00 | 1 | 0.05 | 0.00 | 1 | 0.03 | 0.00 |
| *Ramusella clavipectinata* | 0 | 0.00 | 0.00 | 0 | 0.00 | 0.00 | 1 | 0.09 | 0.00 | 3 | 0.05 | 0.00 | 4 | 0.05 | 0.00 |
| *Ramusella furcata* | 0 | 0.00 | 0.00 | 0 | 0.00 | 0.00 | 0 | 0.00 | 0.00 | 1 | 0.05 | 0.00 | 1 | 0.03 | 0.00 |
| *Scheloribates latipes* | 0 | 0.00 | 0.00 | 0 | 0.00 | 0.00 | 0 | 0.00 | 0.00 | 2 | 0.11 | 0.00 | 2 | 0.05 | 0.00 |
| *Scheloribates pallidulus* | 0 | 0.00 | 0.00 | 0 | 0.00 | 0.00 | 3 | 0.18 | 0.00 | 9 | 0.26 | 0.00 | 12 | 0.18 | 0.00 |
| *Spatiodamaeus boreus* | 0 | 0.00 | 0.00 | 0 | 0.00 | 0.00 | 0 | 0.00 | 0.00 | 1 | 0.05 | 0.00 | 1 | 0.03 | 0.00 |
| *Steganacarus* (*Steganacarus*) *magnus* | 1 | 0.25 | 0.01 | 0 | 0.00 | 0.00 | 1 | 0.09 | 0.00 | 0 | 0.00 | 0.00 | 2 | 0.05 | 0.00 |
| *Steganacarus* (*Tropacarus*) *carinatus* | 0 | 0.00 | 0.00 | 0 | 0.00 | 0.00 | 0 | 0.00 | 0.00 | 2 | 0.05 | 0.00 | 2 | 0.03 | 0.00 |
| *Subiasella quadrimaculata* | 0 | 0.00 | 0.00 | 0 | 0.00 | 0.00 | 0 | 0.00 | 0.00 | 6 | 0.21 | 0.00 | 6 | 0.10 | 0.00 |
| *Suctobelba atomaria* | 0 | 0.00 | 0.00 | 0 | 0.00 | 0.00 | 0 | 0.00 | 0.00 | 2 | 0.05 | 0.00 | 2 | 0.03 | 0.00 |
| *Tectocepheus* sp. | 1 | 0.25 | 0.01 | 0 | 0.00 | 0.00 | 0 | 0.00 | 0.00 | 0 | 0.00 | 0.00 | 1 | 0.03 | 0.00 |
| *Tectocepheus velatus alatus* | 0 | 0.00 | 0.00 | 0 | 0.00 | 0.00 | 1 | 0.09 | 0.00 | 2 | 0.05 | 0.00 | 3 | 0.05 | 0.00 |
| *Xenillus tegeocranus* | 0 | 0.00 | 0.00 | 0 | 0.00 | 0.00 | 0 | 0.00 | 0.00 | 1 | 0.05 | 0.00 | 1 | 0.03 | 0.00 |
| *Zygoribatula exilis* | 0 | 0.00 | 0.00 | 0 | 0.00 | 0.00 | 0 | 0.00 | 0.00 | 2 | 0.11 | 0.00 | 2 | 0.05 | 0.00 |
|  | **KNP 1 DD** | | | **KNP 2 DD** | | | **KNP 3 DD** | | | **KNP 4 DD** | | | **KNP Total** | | |
| Species | Abu | Fre | Dom | Abu | Fre | Dom | Abu | Fre | Dom | Abu | Fre | Dom | Abu | Fre | Dom |
| **Acari, Mesostigmata** | 141 | 0.91 | 0.23 | 162 | 1.00 | 0.10 | 272 | 0.91 | 0.05 | 381 | 0.91 | 0.06 | 956 | 0.92 | 0.07 |
| *Dendrolaelaps cornutus* | 5 | 0.18 | 0.01 | 9 | 0.29 | 0.01 | 8 | 0.27 | 0.00 | 21 | 0.18 | 0.00 | 43 | 0.23 | 0.00 |
| *Dendrolaelaps pini* | 0 | 0.00 | 0.00 | 26 | 0.14 | 0.02 | 89 | 0.27 | 0.02 | 9 | 0.27 | 0.00 | 124 | 0.18 | 0.01 |
| *Dendrolaelaps* sp. | 0 | 0.00 | 0.00 | 0 | 0.00 | 0.00 | 4 | 0.09 | 0.00 | 3 | 0.18 | 0.00 | 7 | 0.08 | 0.00 |
| *Dendrolaelaps zwoelferi* | 0 | 0.00 | 0.00 | 0 | 0.00 | 0.00 | 0 | 0.00 | 0.00 | 5 | 0.18 | 0.00 | 5 | 0.05 | 0.00 |
| *Dermanyssus gallinae* | 0 | 0.00 | 0.00 | 0 | 0.00 | 0.00 | 0 | 0.00 | 0.00 | 1 | 0.09 | 0.00 | 1 | 0.03 | 0.00 |
| *Gamasellodes bicolor* | 0 | 0.00 | 0.00 | 0 | 0.00 | 0.00 | 0 | 0.00 | 0.00 | 13 | 0.09 | 0.00 | 13 | 0.03 | 0.00 |
| *Gamasellus montanus* | 2 | 0.09 | 0.00 | 0 | 0.00 | 0.00 | 4 | 0.18 | 0.00 | 3 | 0.18 | 0.00 | 9 | 0.13 | 0.00 |
| *Geholaspis longispinosus* | 0 | 0.00 | 0.00 | 0 | 0.00 | 0.00 | 1 | 0.09 | 0.00 | 0 | 0.00 | 0.00 | 1 | 0.03 | 0.00 |
| *Geholaspis mandibularis* | 1 | 0.09 | 0.00 | 2 | 0.14 | 0.00 | 0 | 0.00 | 0.00 | 0 | 0.00 | 0.00 | 3 | 0.05 | 0.00 |
| *Holoparasitus* sp. | 1 | 0.09 | 0.00 | 0 | 0.00 | 0.00 | 0 | 0.00 | 0.00 | 0 | 0.00 | 0.00 | 1 | 0.03 | 0.00 |
| *Hoploseius oblongus* | 126 | 0.55 | 0.21 | 0 | 0.00 | 0.00 | 1 | 0.09 | 0.00 | 0 | 0.00 | 0.00 | 127 | 0.18 | 0.01 |
| *Lasioseius muricatus* | 0 | 0.00 | 0.00 | 0 | 0.00 | 0.00 | 1 | 0.09 | 0.00 | 0 | 0.00 | 0.00 | 1 | 0.03 | 0.00 |
| *Lasioseius ometes* | 0 | 0.00 | 0.00 | 3 | 0.29 | 0.00 | 0 | 0.00 | 0.00 | 1 | 0.09 | 0.00 | 4 | 0.08 | 0.00 |
| *Lasioseius zerconoides* | 3 | 0.18 | 0.00 | 33 | 0.43 | 0.02 | 25 | 0.36 | 0.00 | 1 | 0.09 | 0.00 | 62 | 0.25 | 0.00 |
| *Lysigamasus runcatellus* | 0 | 0.00 | 0.00 | 0 | 0.00 | 0.00 | 0 | 0.00 | 0.00 | 1 | 0.09 | 0.00 | 1 | 0.03 | 0.00 |
| Parasitidae | 0 | 0.00 | 0.00 | 3 | 0.14 | 0.00 | 3 | 0.18 | 0.00 | 19 | 0.36 | 0.00 | 25 | 0.18 | 0.00 |
| *Parasitus* sp. | 0 | 0.00 | 0.00 | 0 | 0.00 | 0.00 | 0 | 0.00 | 0.00 | 4 | 0.18 | 0.00 | 4 | 0.05 | 0.00 |
| *Pergamasus rühmi* | 0 | 0.00 | 0.00 | 0 | 0.00 | 0.00 | 10 | 0.27 | 0.00 | 4 | 0.09 | 0.00 | 14 | 0.10 | 0.00 |
| *Pergamasus* sp. | 0 | 0.00 | 0.00 | 1 | 0.14 | 0.00 | 15 | 0.27 | 0.00 | 3 | 0.27 | 0.00 | 19 | 0.18 | 0.00 |
| *Porrhostaspis lunulata* | 0 | 0.00 | 0.00 | 0 | 0.00 | 0.00 | 1 | 0.09 | 0.00 | 0 | 0.00 | 0.00 | 1 | 0.03 | 0.00 |
| *Thenargamasus* sp. | 0 | 0.00 | 0.00 | 3 | 0.29 | 0.00 | 0 | 0.00 | 0.00 | 0 | 0.00 | 0.00 | 3 | 0.05 | 0.00 |
| *Trachytes aegrota* | 0 | 0.00 | 0.00 | 8 | 0.14 | 0.00 | 14 | 0.27 | 0.00 | 8 | 0.18 | 0.00 | 30 | 0.15 | 0.00 |
| *Trichouropoda sociata* | 0 | 0.00 | 0.00 | 0 | 0.00 | 0.00 | 0 | 0.00 | 0.00 | 1 | 0.09 | 0.00 | 1 | 0.03 | 0.00 |
| *Trichouropoda structura* | 0 | 0.00 | 0.00 | 4 | 0.14 | 0.00 | 0 | 0.00 | 0.00 | 0 | 0.00 | 0.00 | 4 | 0.03 | 0.00 |
| *Veigaia kochi* | 0 | 0.00 | 0.00 | 1 | 0.14 | 0.00 | 0 | 0.00 | 0.00 | 0 | 0.00 | 0.00 | 1 | 0.03 | 0.00 |
| *Veigaia nemorensis* | 0 | 0.00 | 0.00 | 0 | 0.00 | 0.00 | 2 | 0.18 | 0.00 | 0 | 0.00 | 0.00 | 2 | 0.05 | 0.00 |
| *Veigaia* sp. | 0 | 0.00 | 0.00 | 0 | 0.00 | 0.00 | 1 | 0.09 | 0.00 | 0 | 0.00 | 0.00 | 1 | 0.03 | 0.00 |
| *Veigaia transisale* | 0 | 0.00 | 0.00 | 0 | 0.00 | 0.00 | 1 | 0.09 | 0.00 | 0 | 0.00 | 0.00 | 1 | 0.03 | 0.00 |
| *Zercon curiosus* | 0 | 0.00 | 0.00 | 1 | 0.14 | 0.00 | 0 | 0.00 | 0.00 | 0 | 0.00 | 0.00 | 1 | 0.03 | 0.00 |
| *Zercon schweizeri* | 0 | 0.00 | 0.00 | 0 | 0.00 | 0.00 | 0 | 0.00 | 0.00 | 80 | 0.18 | 0.01 | 80 | 0.05 | 0.01 |
| *Zercon storkani* | 0 | 0.00 | 0.00 | 4 | 0.14 | 0.00 | 15 | 0.45 | 0.00 | 67 | 0.18 | 0.01 | 86 | 0.20 | 0.01 |
| *Zerconopsis michaeli* | 1 | 0.09 | 0.00 | 24 | 0.14 | 0.01 | 0 | 0.00 | 0.00 | 0 | 0.00 | 0.00 | 25 | 0.05 | 0.00 |
| *Zerconopsis remiger* | 2 | 0.18 | 0.00 | 40 | 0.71 | 0.02 | 77 | 0.55 | 0.01 | 137 | 0.55 | 0.02 | 256 | 0.48 | 0.02 |
| **Acari, Oribatida** | 462 | 0.91 | 0.77 | 1510 | 1.00 | 0.90 | 5488 | **1.00** | 0.95 | 5496 | **1.00** | 0.94 | 12956 | 0.98 | 0.93 |
| *Adoristes ovatus* | 0 | 0.00 | 0.00 | 0 | 0.00 | 0.00 | 2 | 0.18 | 0.00 | 3 | 0.18 | 0.00 | 5 | 0.10 | 0.00 |
| *Autogneta longilamellata* | 0 | 0.00 | 0.00 | 0 | 0.00 | 0.00 | 2 | 0.18 | 0.00 | 0 | 0.00 | 0.00 | 2 | 0.05 | 0.00 |
| *Belba corynopus* | 0 | 0.00 | 0.00 | 3 | 0.14 | 0.00 | 1 | 0.09 | 0.00 | 1 | 0.09 | 0.00 | 5 | 0.08 | 0.00 |
| *Berniniella sigma* | 0 | 0.00 | 0.00 | 0 | 0.00 | 0.00 | 0 | 0.00 | 0.00 | 1 | 0.09 | 0.00 | 1 | 0.03 | 0.00 |
| *Caleremaeus monilipes* | 1 | 0.09 | 0.00 | 0 | 0.00 | 0.00 | 0 | 0.00 | 0.00 | 1 | 0.09 | 0.00 | 2 | 0.05 | 0.00 |
| *Carabodes areolatus* | 82 | 0.64 | 0.14 | 110 | 0.86 | 0.07 | 585 | 1.00 | 0.10 | 253 | 0.91 | 0.04 | 1030 | 0.85 | 0.07 |
| *Carabodes coriaceus* | 11 | 0.18 | 0.02 | 5 | 0.14 | 0.00 | 18 | 0.36 | 0.00 | 112 | 0.36 | 0.02 | 146 | 0.28 | 0.01 |
| *Carabodes femoralis* | 228 | 0.91 | 0.38 | 1178 | 1.00 | 0.70 | 4531 | 1.00 | 0.79 | 4685 | 1.00 | 0.80 | 10622 | 0.98 | 0.76 |
| *Carabodes labyrinthicus* | 17 | 0.36 | 0.03 | 7 | 0.29 | 0.00 | 24 | 0.64 | 0.00 | 43 | 0.73 | 0.01 | 91 | 0.53 | 0.01 |
| *Carabodes ornatus* | 10 | 0.36 | 0.02 | 1 | 0.14 | 0.00 | 3 | 0.18 | 0.00 | 5 | 0.18 | 0.00 | 19 | 0.23 | 0.00 |
| *Carabodes reticulatus* | 8 | 0.27 | 0.01 | 4 | 0.29 | 0.00 | 27 | 0.36 | 0.00 | 23 | 0.45 | 0.00 | 62 | 0.35 | 0.00 |
| *Carabodes* sp. | 3 | 0.18 | 0.00 | 96 | 0.29 | 0.06 | 2 | 0.18 | 0.00 | 0 | 0.00 | 0.00 | 101 | 0.15 | 0.01 |
| *Carabodes tenuis* | 0 | 0.00 | 0.00 | 0 | 0.00 | 0.00 | 8 | 0.27 | 0.00 | 11 | 0.27 | 0.00 | 19 | 0.15 | 0.00 |
| *Cepheus cepheiformis* | 1 | 0.09 | 0.00 | 0 | 0.00 | 0.00 | 23 | 0.45 | 0.00 | 28 | 0.55 | 0.00 | 52 | 0.30 | 0.00 |
| *Cepheus dentatus* | 1 | 0.09 | 0.00 | 7 | 0.57 | 0.00 | 14 | 0.36 | 0.00 | 3 | 0.18 | 0.00 | 25 | 0.28 | 0.00 |
| *Cepheus* sp. | 0 | 0.00 | 0.00 | 1 | 0.14 | 0.00 | 9 | 0.18 | 0.00 | 2 | 0.09 | 0.00 | 12 | 0.10 | 0.00 |
| *Ceratoppia bipilis* | 0 | 0.00 | 0.00 | 0 | 0.00 | 0.00 | 1 | 0.09 | 0.00 | 0 | 0.00 | 0.00 | 1 | 0.03 | 0.00 |
| *Ceratoppia quadridentata* | 1 | 0.09 | 0.00 | 0 | 0.00 | 0.00 | 0 | 0.00 | 0.00 | 0 | 0.00 | 0.00 | 1 | 0.03 | 0.00 |
| *Chamobates borealis* | 3 | 0.18 | 0.00 | 1 | 0.14 | 0.00 | 59 | 0.45 | 0.01 | 20 | 0.45 | 0.00 | 83 | 0.33 | 0.01 |
| *Chamobates spinosus* | 1 | 0.09 | 0.00 | 1 | 0.14 | 0.00 | 0 | 0.00 | 0.00 | 0 | 0.00 | 0.00 | 2 | 0.05 | 0.00 |
| *Chamobates voigtsi* | 0 | 0.00 | 0.00 | 1 | 0.14 | 0.00 | 0 | 0.00 | 0.00 | 1 | 0.09 | 0.00 | 2 | 0.05 | 0.00 |
| *Autogneta dalecarlica* | 0 | 0.00 | 0.00 | 2 | 0.14 | 0.00 | 7 | 0.09 | 0.00 | 0 | 0.00 | 0.00 | 9 | 0.05 | 0.00 |
| *Cymbaeremaeus cymba* | 0 | 0.00 | 0.00 | 0 | 0.00 | 0.00 | 1 | 0.09 | 0.00 | 0 | 0.00 | 0.00 | 1 | 0.03 | 0.00 |
| *Damaeus* (*Paradamaeus*) *clavipes* | 0 | 0.00 | 0.00 | 0 | 0.00 | 0.00 | 0 | 0.00 | 0.00 | 2 | 0.18 | 0.00 | 2 | 0.05 | 0.00 |
| *Damaeus riparius* | 0 | 0.00 | 0.00 | 3 | 0.14 | 0.00 | 1 | 0.09 | 0.00 | 5 | 0.27 | 0.00 | 9 | 0.13 | 0.00 |
| *Dissorhina ornata* | 0 | 0.00 | 0.00 | 1 | 0.14 | 0.00 | 3 | 0.09 | 0.00 | 0 | 0.00 | 0.00 | 4 | 0.05 | 0.00 |
| *Eueremaus oblongus* | 0 | 0.00 | 0.00 | 0 | 0.00 | 0.00 | 0 | 0.00 | 0.00 | 2 | 0.09 | 0.00 | 2 | 0.03 | 0.00 |
| *Euphthiracarus cribrarius* | 0 | 0.00 | 0.00 | 0 | 0.00 | 0.00 | 3 | 0.09 | 0.00 | 0 | 0.00 | 0.00 | 3 | 0.03 | 0.00 |
| *Lagenobates lagenulus* | 0 | 0.00 | 0.00 | 1 | 0.14 | 0.00 | 13 | 0.18 | 0.00 | 1 | 0.09 | 0.00 | 15 | 0.10 | 0.00 |
| *Liacarus coracinus* | 2 | 0.09 | 0.00 | 0 | 0.00 | 0.00 | 3 | 0.18 | 0.00 | 1 | 0.09 | 0.00 | 6 | 0.10 | 0.00 |
| *Liebstadia longior* | 1 | 0.09 | 0.00 | 0 | 0.00 | 0.00 | 3 | 0.18 | 0.00 | 1 | 0.09 | 0.00 | 5 | 0.10 | 0.00 |
| *Liebstadia pannonica* | 3 | 0.18 | 0.00 | 0 | 0.00 | 0.00 | 4 | 0.27 | 0.00 | 1 | 0.09 | 0.00 | 8 | 0.15 | 0.00 |
| *Liebstadia similis* | 1 | 0.09 | 0.00 | 0 | 0.00 | 0.00 | 0 | 0.00 | 0.00 | 0 | 0.00 | 0.00 | 1 | 0.03 | 0.00 |
| *Minunthozetes pseudofusiger* | 0 | 0.00 | 0.00 | 0 | 0.00 | 0.00 | 0 | 0.00 | 0.00 | 3 | 0.09 | 0.00 | 3 | 0.03 | 0.00 |
| *Nanhermannia* cf. *coronata* | 2 | 0.09 | 0.00 | 8 | 0.14 | 0.00 | 0 | 0.00 | 0.00 | 2 | 0.18 | 0.00 | 12 | 0.10 | 0.00 |
| *Nanhermannia comitalis* | 0 | 0.00 | 0.00 | 0 | 0.00 | 0.00 | 4 | 0.18 | 0.00 | 0 | 0.00 | 0.00 | 4 | 0.05 | 0.00 |
| *Nothrus silvestris* | 1 | 0.09 | 0.00 | 0 | 0.00 | 0.00 | 5 | 0.36 | 0.00 | 0 | 0.00 | 0.00 | 6 | 0.13 | 0.00 |
| Oppidae | 0 | 0.00 | 0.00 | 0 | 0.00 | 0.00 | 2 | 0.18 | 0.00 | 1 | 0.09 | 0.00 | 3 | 0.08 | 0.00 |
| *Oppiella* (*Moritzoppia*)*keilbachi* | 0 | 0.00 | 0.00 | 0 | 0.00 | 0.00 | 11 | 0.09 | 0.00 | 2 | 0.09 | 0.00 | 13 | 0.05 | 0.00 |
| *Oppiella* (*Moritzoppia*) *translamellata* | 0 | 0.00 | 0.00 | 0 | 0.00 | 0.00 | 1 | 0.09 | 0.00 | 0 | 0.00 | 0.00 | 1 | 0.03 | 0.00 |
| *Oppiella* (*Moritzoppia*) *unicarinata* | 0 | 0.00 | 0.00 | 0 | 0.00 | 0.00 | 0 | 0.00 | 0.00 | 2 | 0.09 | 0.00 | 2 | 0.03 | 0.00 |
| *Oppiella* (*Oppiella*) *nova* | 0 | 0.00 | 0.00 | 2 | 0.14 | 0.00 | 0 | 0.00 | 0.00 | 0 | 0.00 | 0.00 | 2 | 0.03 | 0.00 |
| *Oppiella* (*Rhinoppia*) *subpectinata* | 0 | 0.00 | 0.00 | 0 | 0.00 | 0.00 | 1 | 0.09 | 0.00 | 0 | 0.00 | 0.00 | 1 | 0.03 | 0.00 |
| *Oribatella calcarata* | 15 | 0.36 | 0.02 | 35 | 0.57 | 0.02 | 10 | 0.55 | 0.00 | 159 | 0.55 | 0.03 | 219 | 0.50 | 0.02 |
| *Oribatella quadricornuta* | 0 | 0.00 | 0.00 | 23 | 0.14 | 0.01 | 11 | 0.18 | 0.00 | 41 | 0.27 | 0.01 | 75 | 0.15 | 0.01 |
| *Oribatula tibialis* | 0 | 0.00 | 0.00 | 0 | 0.00 | 0.00 | 0 | 0.00 | 0.00 | 1 | 0.09 | 0.00 | 1 | 0.03 | 0.00 |
| other juveniles | 5 | 0.18 | 0.01 | 3 | 0.29 | 0.00 | 1 | 0.09 | 0.00 | 2 | 0.18 | 0.00 | 11 | 0.18 | 0.00 |
| *Phthiracarus bryobius* | 0 | 0.00 | 0.00 | 0 | 0.00 | 0.00 | 4 | 0.18 | 0.00 | 7 | 0.09 | 0.00 | 11 | 0.08 | 0.00 |
| *Phthiracarus longulus* | 3 | 0.27 | 0.00 | 8 | 0.43 | 0.00 | 35 | 0.64 | 0.01 | 15 | 0.45 | 0.00 | 61 | 0.45 | 0.00 |
| *Platynothrus peltifer* | 0 | 0.00 | 0.00 | 0 | 0.00 | 0.00 | 4 | 0.09 | 0.00 | 4 | 0.18 | 0.00 | 8 | 0.08 | 0.00 |
| *Ramusella clavipectinata* | 2 | 0.18 | 0.00 | 0 | 0.00 | 0.00 | 7 | 0.18 | 0.00 | 8 | 0.27 | 0.00 | 17 | 0.18 | 0.00 |
| *Ramusella insculpta* | 0 | 0.00 | 0.00 | 0 | 0.00 | 0.00 | 2 | 0.09 | 0.00 | 0 | 0.00 | 0.00 | 2 | 0.03 | 0.00 |
| *Scheloribates* (*Hemileius*) *initialis* | 0 | 0.00 | 0.00 | 1 | 0.14 | 0.00 | 2 | 0.09 | 0.00 | 1 | 0.09 | 0.00 | 4 | 0.08 | 0.00 |
| *Scheloribates pallidulus* | 38 | 0.64 | 0.06 | 6 | 0.29 | 0.00 | 29 | 0.45 | 0.01 | 9 | 0.36 | 0.00 | 82 | 0.45 | 0.01 |
| *Scheloribates* sp. | 1 | 0.09 | 0.00 | 0 | 0.00 | 0.00 | 0 | 0.00 | 0.00 | 0 | 0.00 | 0.00 | 1 | 0.03 | 0.00 |
| *Siculobata leontonycha* | 0 | 0.00 | 0.00 | 0 | 0.00 | 0.00 | 3 | 0.09 | 0.00 | 0 | 0.00 | 0.00 | 3 | 0.03 | 0.00 |
| *Spatiodamaeus boreus* | 0 | 0.00 | 0.00 | 0 | 0.00 | 0.00 | 1 | 0.09 | 0.00 | 1 | 0.09 | 0.00 | 2 | 0.05 | 0.00 |
| *Steganacarus* (*Atropacarus*) *striculus* | 0 | 0.00 | 0.00 | 2 | 0.29 | 0.00 | 7 | 0.18 | 0.00 | 33 | 0.27 | 0.01 | 42 | 0.18 | 0.00 |
| *Tectocepheus* sp. | 1 | 0.09 | 0.00 | 0 | 0.00 | 0.00 | 0 | 0.00 | 0.00 | 0 | 0.00 | 0.00 | 1 | 0.03 | 0.00 |
| *Tectocepheus velatus alatus* | 3 | 0.18 | 0.00 | 0 | 0.00 | 0.00 | 1 | 0.09 | 0.00 | 0 | 0.00 | 0.00 | 4 | 0.08 | 0.00 |
| *Tectocepheus velatus velatus* | 5 | 0.09 | 0.01 | 0 | 0.00 | 0.00 | 0 | 0.00 | 0.00 | 0 | 0.00 | 0.00 | 5 | 0.03 | 0.00 |
| *Zygoribatula exilis* | 12 | 0.18 | 0.02 | 0 | 0.00 | 0.00 | 0 | 0.00 | 0.00 | 0 | 0.00 | 0.00 | 12 | 0.05 | 0.00 |

**Appendix** Mites (Acari: Mesostigmata, Oribatida) occuring on fruiting bodies of *Fomitopsis pinicola* in Białowieża National Park (BNP) and Karkonosze National Park (KNP), Poland. Abu – abundance. Fre – frequency. Dom – Dominance.
